# Extracting True Virus SERS Spectra and Augmenting Data for Improved Virus Classification and Quantification

**DOI:** 10.1021/acssensors.4c03397

**Published:** 2025-05-18

**Authors:** Yufang Liu, Yanjun Yang, Haoran Lu, Jiaheng Cui, Xianyan Chen, Ping Ma, Wenxuan Zhong, Yiping Zhao

**Affiliations:** † Department of Statistics, Franklin College of Arts and Sciences, 1355 University of Georgia, Athens, Georgia 30602, United States; ‡ Department of Physics and Astronomy, Franklin College of Arts and Sciences, University of Georgia, Athens, Georgia 30602, United States; § School of Electrical and Computer Engineering, College of Engineering, The University of Georgia, Athens, Georgia 30602, United States; ∥ Department of Epidemiology & Biostatistics, College of Public Health, The University of Georgia, Athens, Georgia 30602, United States

**Keywords:** surface-enhanced Raman spectroscopy, true spectrum extraction, data augmentation, machine learning, virus detection

## Abstract

Surface-enhanced Raman spectroscopy (SERS) is a transformative tool for infectious disease diagnostics, offering rapid and sensitive species identification. However, background spectra in biological samples complicate analyte peak detection, increase the limit of detection, and hinder data augmentation. To address these challenges, we developed a deep learning framework utilizing dual neural networks to extract true virus SERS spectra and estimate concentration coefficients in water for 12 different respiratory viruses. The extracted spectra showed a high similarity to those obtained at the highest viral concentration, validating their accuracy. Using these spectra and the derived concentration coefficients, we augmented spectral data sets across varying virus concentrations in water. XGBoost models trained on these augmented data sets achieved overall classification and concentration prediction accuracy of 92.3% with a coefficient of determination (*R* ^2^) > 0.95. Additionally, the extracted spectra and coefficients were used to augment data sets in saliva backgrounds. When tested against real virus-in-saliva spectra, the augmented spectra-trained XGBoost models achieved 91.9% accuracy in classification and concentration prediction with *R* ^2^ > 0.9, demonstrating the robustness of the approach. By delivering clean and uncontaminated spectra, this methodology can significantly improve species identification, differentiation, and quantification and advance SERS-based detection and diagnostics.

## Graphical abstract

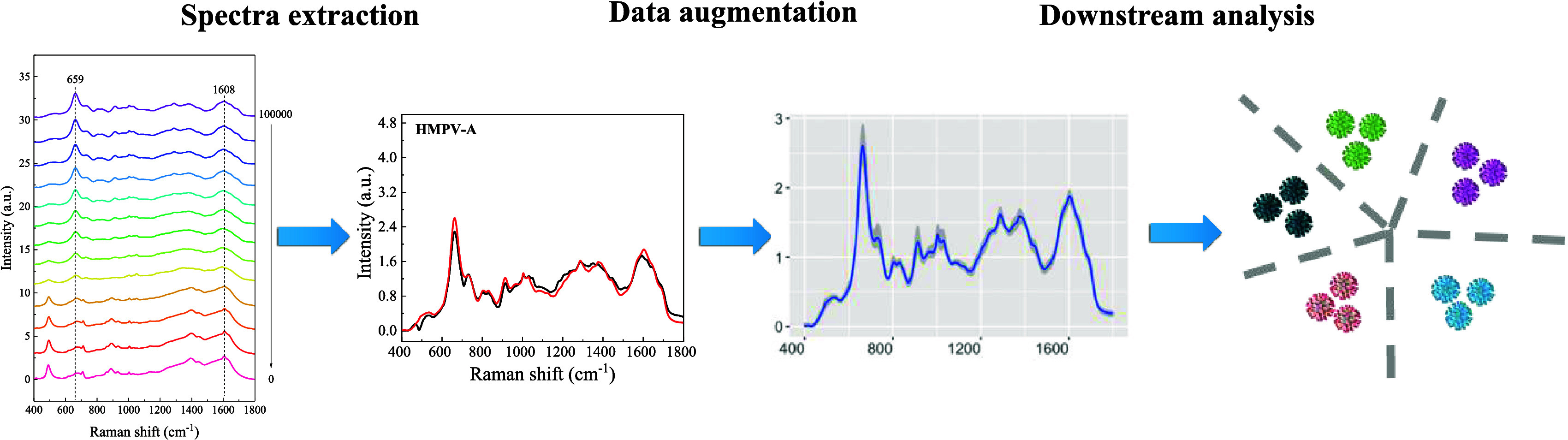

## Introduction

Surface-enhanced Raman spectroscopy (SERS) emerges as a cutting-edge technology poised to revolutionize chemical and biological detection and medical diagnostics, owing to its high sensitivity, molecular fingerprint spectrum, ease of operation, and point-of-care compatibility.^1^ However, in many biological detections, the true spectrum of a target analyte is buried in interference spectra of the buffer, medium, or background. For example, in clinical applications such as diagnosis of viral or bacterial infections, specimens are usually taken from human nasal swabs, saliva, blood, etc.,^2,3^ and in order to prevent any possible transmission of the diseases, the specimens are usually inactivated via inactivation medium.^4^ Both the body fluids and the inactivation solutions could contain molecules that can access SERS hot spots and produce SERS signatures that do not belong to the target analytes. Even when the analyte is made from the buffer solution, when the analyte concentration increases, the buffer/medium background could still have an effect on the resulting spectra if the molecules from the buffer or medium have high affinity to SERS substrates.^5,6^ These background interference spectra introduce three challenges in the subsequent SERS spectrum analysis. First, it prevents the accurate determination of characteristic SERS peaks for the target analyte, thereby impacting the accurate identification of the analyte via its characteristic vibrational modes. Second, the existence of the background spectrum contributes to a larger limit of detection (LOD). Last, the presence of background spectra hampers effective data augmentation, as some analytes may be present in diverse background media.

In addition, recently machine learning (ML) has been used to develop robust and accurate diagnostic models based on SERS spectra.^7–9^ Due to the presence of noise, background interference, and overlapping signals from multiple analytes, the interpretation of SERS spectra can be complex. ML algorithms, such as support vector machines,^10–14^ deep learning (DL),^15–18^ as well as regression models based on these algorithms,^10,11,19–21^ can automatically extract meaningful features from SERS spectra, discriminate between different analytes or biomarkers, classify samples into distinct categories (e.g., healthy vs diseased), and make prediction for analyte concentrations, leading to improved diagnostic accuracy and efficiency. However, for clinical diagnostics, some challenges remain for the SERS + ML strategy. Especially, clinical data sets in SERS diagnostics are often limited in size due to factors such as cost, time constraints, and patient privacy concerns. By augmenting the SERS data set with variations of the original spectra, such as rotations, translations, and noise addition, a more diverse and representative data set that captures the full range of variability present in real-world samples can be created.^17,22,23^ This helps to improve the robustness and generalization ability of machine learning models trained on the augmented data, making them more effective in handling unseen samples and real-world scenarios. Also, data augmentation can help address the issue of class imbalance, which is commonly encountered in clinical data sets. In medical diagnostics, certain conditions or diseases may be less prevalent than others, leading to imbalanced data sets where the minority class is underrepresented. Augmentation techniques, such as oversampling of minority classes or generating synthetic data for underrepresented classes, can help alleviate this imbalance, leading to more accurate and reliable diagnostic models. Furthermore, data augmentation facilitates the training of machine learning models to be more robust to variations and artifacts present in real-world clinical samples. A good data augmentation strategy requires not only to preserve the underlying characteristics of the SERS spectra and cover a wide range of variations that are representative of the natural variability in the data set but also to have high-quality original spectra with minimal noise, artifacts, or distortions.^24,25^ Low-quality spectra can lead to inaccurate augmentation and may degrade the performance of machine learning models trained on augmented data.

Therefore, it is important to extract the true spectrum of the target analyte from SERS measurements to tackle the aforementioned challenges. In principle, since every SERS spectrum can be viewed as a combination of a SERS spectrum from the target analyte, buffer, medium, or other background, and given the assumption that the spectrum is invariant at different concentrations, if one can measure multiple spectra of the target analyte under different concentrations titrated under the same buffer/medium, and the spectrum from buffer/medium/background can be determined experimentally, one shall be able to mathematically extract the true SERS spectrum from these experimental spectra. In the SERS base literature, though people have not used this strategy to predict the spectra of target analytes, the linear combination has been used to predict the composition of analytes that contribute to the entire SERS spectrum.^26–28^ For example, Abell et al. used the linear combination principle to predict the nucleic acid base ratio during the DNA hybridization process using the changes in SERS spectra.^28^

This work presents a linear decomposition strategy to extract the true SERS spectra of the target viruses. A comprehensive framework leveraging a neural network (NN) is introduced to estimate the true analyte spectra and corresponding concentration coefficients. Using the extracted true virus spectrum (ETVS) and the fluctuations observed in measured spectra, a data augmentation strategy is developed to generate a training data set that mimics virus spectra at various concentrations. Two XGBoost models,^29^ one for differentiation and the other for regression, trained on this augmented data set achieved 92.3% classification accuracy for virus types and excellent concentration regression performance, with an average coefficient of determination *R* ^2^ of 0.986. Such a strategy can be further extended to classify viruses and quantify their concentration in saliva, achieving 91.9% accuracy in virus classification and an *R* ^2^ of 0.965 for concentration regression. These results demonstrate that using extracted true SERS spectra for data augmentation can produce accurate, robust, and reliable outcomes in downstream classification and quantification analyses.

## Model and Spectra Analysis Methods

### Method Overview

The general spectral analysis procedure is shown in Figure 1. The first step involves area normalization of the raw SERS spectra of different viral concentrations and background medium. Assuming that each normalized spectrum is a linear combination of the true virus spectrum (TVS) and that of the background medium, an NN model is employed to extract the corresponding ETVS and concentration coefficients. The ETVS as well as coefficients can be used to construct the augmentation spectral database and feed it into two different XGBoost models, one for virus-type classification and the other for virus concentration regression.

**Figure 1:**
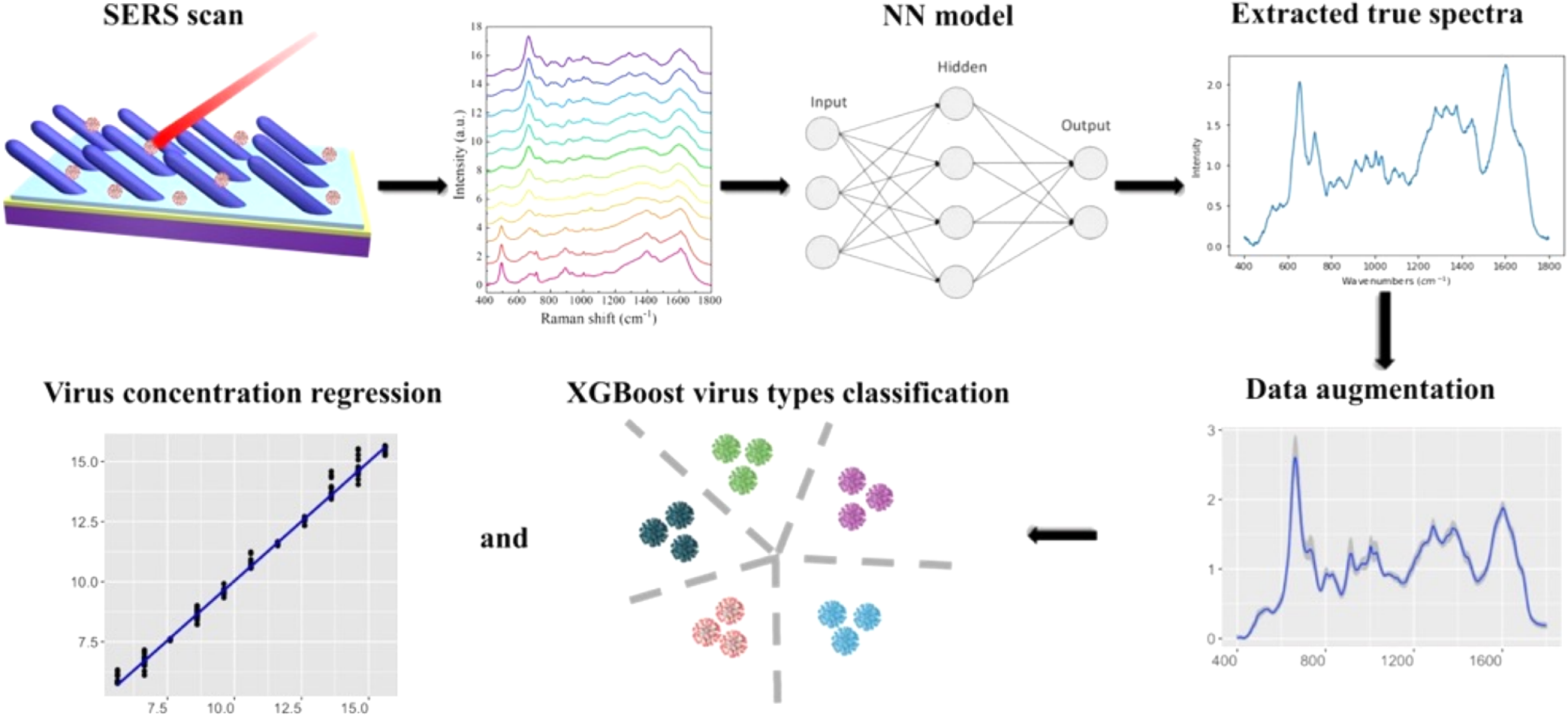
Overview of the analysis procedure for true SERS spectral extraction and data augmentation.

### Source of Virus SERS Spectra

The virus SERS spectra adopted from ref 10, that is, virus spectra of different concentration *c*, with *c* ∈ *C* = {50, ···, 50,000, 100,000 PFU/mL, were measured under two different background media, water and saliva. Thus, there are two sets of virus spectra. For each virus with a specific concentration, around 500 SERS spectra were obtained, and these measured spectra are termed the mixed spectra, that is, spectra from a mixture of true virus and background medium. Our aim is to extract true spectra of 12 viruses, HMPV-A, HMPV-B, CoV-OC43, Flu B, CoV-229E, CoV-NL63, Ad5, SARS-CoV-2 B1, H1N1, RSV-B1, RSV-A2, and H3N2 (the abbreviations of virus names can be found in Section S1 of Supporting Information (SI)), from the concentration-dependent spectra of virus-in-water, to use the obtained ETVS spectra to establish augmented spectral sets to train both classification and regression deep learning models to validate the advantage of the proposed augmentation strategy, and then to implement this augmentation strategy to classify and quantify virus-in-saliva based on experimental SERS spectra. These virus types are denoted as *t* = 1, 2, ···, 12, respectively. Detailed sample preparation and SERS measurement conditions can be found in Sections S2–S4 of SI, or ref 10. The average concentration-dependent SERS spectra of HMPV-A are shown in Figure 2A, and the spectra for the other 11 viruses are shown in Section S5 of SI.

**Figure 2:**
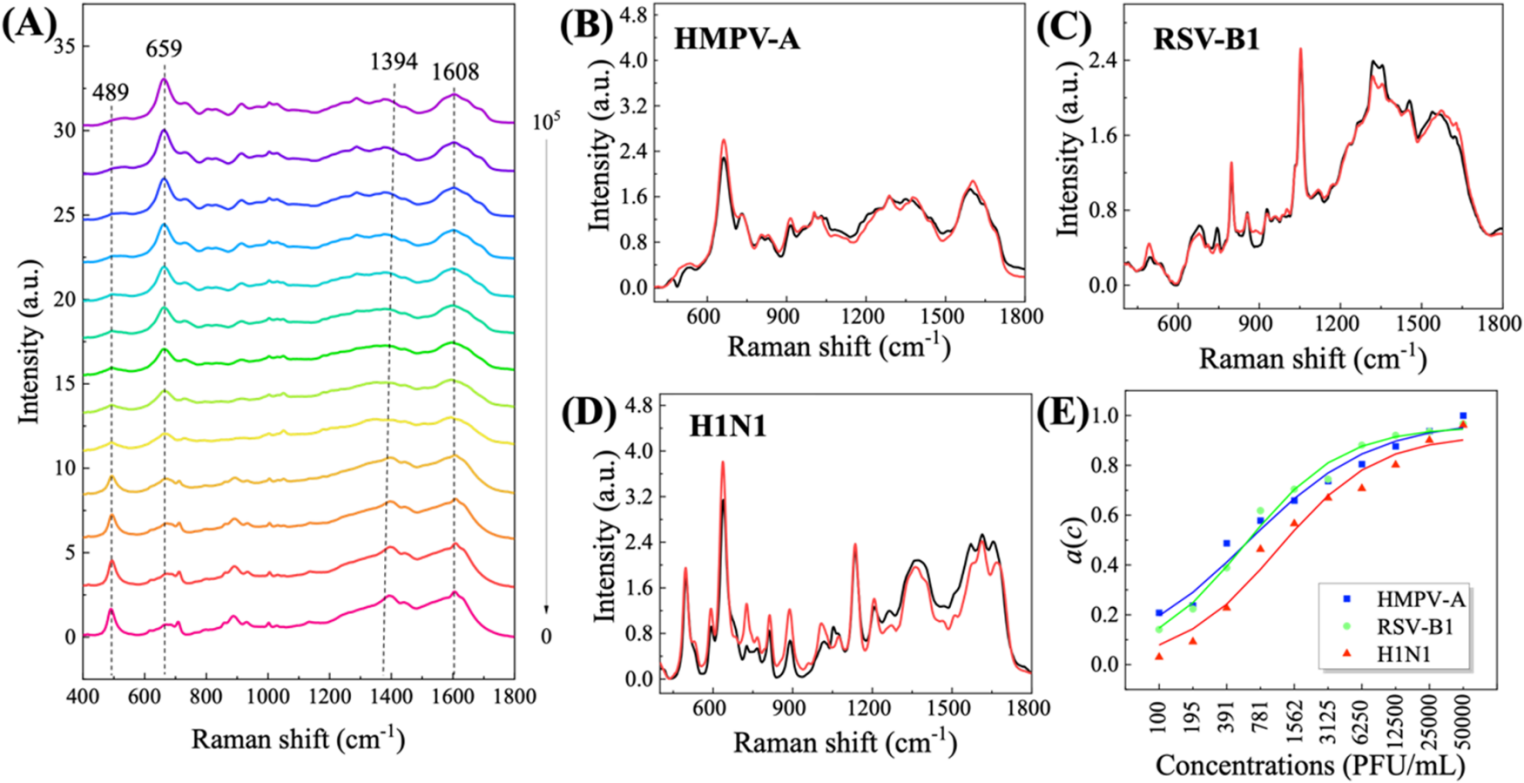
(A) Mean of measured spectra (MMSs) of HMPV-A was obtained at different concentrations. The plots of ETVS and TVS of (B) HMPV-A, (C) RSV-B1, and (D) H1N1. Red curves are ETVS, and black curves are TVS. (E) Extracted concentration coefficients *â*(*c*) versus *c* for three viruses.

### True Virus Spectrum Extraction Strategy

A novel spectral decomposition method is proposed. Let *I*_*c*_(*j*_*c*_, Δν) be the normalized measured spectrum (MS) of a virus at a concentration *c*, where *j*_c_ = 1, 2, ···, *N*_*c*_, denoting each replication of the measurement, with *N*_*c*_ being the total number of measured spectra under the concentration *c* and Δν represents the wavenumber ranging from 400 to 1799 cm^–1^. Mark *I*_M_(*c*, Δν) as the mean of the measured spectrum (MMS) at *c*, *I*_T_(Δν) as the normalized TVS, and *I*_B_(Δν) as the normalized background medium spectrum (BMS). The following equation for spectra decomposition is proposed,^30^

1 $$I_{c}\left(j_{c},\Delta \nu \right)=a\left(c\right)I_{T}\left(\Delta \nu \right)+\left(1-a\left(c\right)\right)I_{B}\left(\Delta \nu \right)+\epsilon \left(\Delta \nu \right)$$

where *a*(*c*) is the linear coefficient, increasing monotonically with *c*, and its value is in the range of 0–1; ϵ­(Δν) is assumed to be a random and independent Gaussian noise in each spectrum. Intuitively, eq 1 decomposes *I*_*c*_ into two main parts, *I*_T_ and *I*_B_, with an additional residual spectrum ϵ­(Δν). When *c* is significantly low (i.e., *a*(*c*) ≪ 1 – *a*(*c*)), the presence of the analyte in the mixture becomes negligible, that is, *I*_*c*_ is dominated by *I*_B_. Conversely, as *c* increases, the influence of *I*_T_ becomes more significant, eventually causing *a*(*c*) to approach 1 when *c* is sufficiently large. Here, it is assumed that *I*_T_(Δν) remains invariant with *c*, as the random distribution of the analyte on the SERS substrate ensures that a sufficient quantity of the analyte is detected in a single SERS measurement.

Both the measured spectrum *I*_*c*_(*j*_*c*_, Δν) and the background spectrum *I*_B_(Δν) can be experimentally determined. Since *I*_*c*_(*j*_*c*_, Δν) can be measured at *M* different concentrations, a least-squares method could, in principle, be used to uniquely determine both *a*(*c*) and *I*_T_(Δν) based on eq 1, as outlined in ref 28. In ref 28, average spectra were utilized to extract the linear coefficients. However, in our experiments, even for the same *c*, noise ϵ­(Δν) significantly affects the results. Using the average spectra and least-squares method to estimate the average *a*(*c*) and *I*_T_(Δν) might be feasible if ϵ­(Δν) represents truly random, independent Gaussian noise and if *N*_*c*_ is sufficiently large.^31,32^ However, statistical tests (see Section S6 of SI) reveal that while the spectral intensities at certain wavenumbers in the normalized spectra exhibit Gaussian and independent behavior, many other wavenumbers deviate from these assumptions, showing non-Gaussian distributions, lack of independence, or both. Consequently, relying on average spectra for predicting *a*(*c*) and *I*_T_(Δν) may not be suitable.^31,32^ To account for the variability in measured spectra, we employed an NN for each virus type to extract ETVS *I*_T_(Δν) and its corresponding coefficient *a*(*c*). Each NN model features a fully connected architecture with rectified linear unit (ReLU) activations, batch normalization, and dropout layers to enhance stability and regularization. The NN’s strength lies in its ability to learn complex data relationships, enabling it to identify intricate patterns and features in SERS spectra effectively.

The inputs for each NN are the wavenumbers, concentrations, BMS, and MMS, and outputs have two branches, one dedicated to the ETVS *Î*_T_ and the other to predicted concentration coefficients *â*(*c*). A six-layer fully connected architecture with ReLU activations (details see Section S7 of SI)^33^ is applied to facilitate the extraction of intricate patterns within the input data. Batch normalization layers are strategically positioned after each linear layer to enhance the training stability and speed. Following the batch normalization layers, a dropout layer with a dropout rate set at 0.2 is incorporated, effectively introducing regularization to mitigate overfitting. The NN is trained by minimizing the following loss function:

2 $$L=\sum_{c\in C}\sum_{\Delta \nu =400}^{1799}{\left(I_{M}\left(c,\Delta \nu \right)-â\left(c\right){Î}_{T}\left(\Delta \nu \right)-\left(1-â\left(c\right)\right)I_{B}\left(\Delta \nu \right)\right)}^{2}$$

where *C* denotes the total number of concentrations, with *c* ∈ *C* = {50, ···, 50,000}. Equation 2 is essentially a mean squared error (MSE) loss that quantifies the goodness of fit of the model. Using this model, the estimation of ETVS *Î*_T_(Δν) for one virus, concentration coefficients *â*(*c*), as well as the prediction for MMS *Î*_M_(*c*, Δν) are obtained. The *â*(*c*) could be used to quantify the relationship of concentration and the proportion of *Î*_T_(Δν) in *Î*_M_. *Î*_T_(Δν) can be used to explore downstream analyses such as virus-type differentiation and concentration regression. The model can be validated based on the results of downstream analysis. In this process, a data augmentation is formulated to expand *Î*_T_(Δν) to a larger database.

### Validating the Results via a Data Augmentation Method

The basis of the data augmentation method is to mimic variation in different measurements by adding realistic residuals to the ETVS to obtain individual predictions for MS. Such a process is carried out by obtaining the shuffled residue spectra. First, the residuals based on both measured *I*_*c*_(*j*_*c*_, Δν) and predicted MMS *Î*_M_(*c*, Δν) at concentration level *c* were obtained, and the estimated residual spectrum *Î*_*c*_^R^(*j*_*c*_, Δν) is calculated as

3 $${Î}_{c}^{R}\left(j_{c},\Delta \nu \right)=I_{c}\left(j_{c},\Delta \nu \right)-{Î}_{M}\left(c,\Delta \nu \right)$$

A total of seven *N*_*c*_ residue spectra are obtained. Based on these residue spectra, shuffled residue spectra *I*_*c*_^SFR^(*j*_*c*_, Δν) are produced, that is, pick up a specific *Î*_*c*_^R^(*j*_*c*_0__, Δν), select a fixed Δν_*i*_, and replace *Î*_*c*_^R^(*j*_*c*_0__, Δν_*i*_) value with a randomly selected value *Î*_*c*_^R^(*j*_*c*_*k*__, Δν_*i*_); repeat the above steps for all the wavenumbers, Δν = 400, ···, 1799 cm^–1^ (a total of *N*_0_ wavenumbers) in the spectrum, then one *I*_*c*_^SFR^(*j*_*c*_, Δν) is obtained. Therefore, we can produce a total of (*N*_*c*_)^*N*_0_^ shuffled residue spectra. Then, the augmented MS *I*_aug_*c*__(*j*_*c*_, Δν) can be written as

4 $$I_{\mathrm{au}g_{c}}\left(j_{c},\Delta \nu \right)=I_{c}^{\mathrm{SFR}}\left(j_{c},\Delta \nu \right)+{Î}_{M}\left(c,\Delta \nu \right)$$

The data augmentation procedure for one concentration *c* within a particular virus type has been implemented, and *N*_*c*_^′^ spectra with *I*_aug_*c*__(*j*_*c*_, Δν), *j*_*c*_ = 1, ···, *N*_*c*_^′^ are obtained. Replicate the same steps for all concentrations *c* ∈ *C* within the specific virus type and obtain ∑_*c*∈*C*_ *N*_*c*_^′^ spectra with *I*_aug_*c*__(*j*_*c*_, Δν), *j*_*c*_ = 1, ···, *N*_*c*_^′^, *c* ∈ *C*, and subsequently, iterate through each virus type *t* = 1, ···, 12 and apply the data augmentation procedure for all concentrations within that type.

It is worth noting that the aim is to use cross-validation techniques in the downstream classification analysis, so within each virus type *t* and concentration *c*, random sampling for data partition is performed, which divides the *N*_*c*_^′^ data set into 80% for training data set and 20% for testing. This approach ensures a robust evaluation of our model’s performance by using distinct data sets for training and testing.

### XGBoost Model for Classification and Regression

The validation process of our method involves conducting downstream analysis of both classification and regression tasks. The premise is that favorable outcomes in these analyses provide compelling evidence for the effectiveness of the model and the data augmentation technique used. The XGBoost model is used for the downstream analysis.^29^ The XGBoost is a well-accepted model in virus-type identification.^34^ It is capable of capturing complex nonlinear relationships between features and the target variables. It is optimized for parallel computing and can handle large data sets efficiently. Taking input as all augmented spectra in different concentrations with different virus types, the XGBoost model would predict the concentration value in a fixed virus type for regression task or predict the classification label for multivirus classification tasks. In our implementation, Python 3.9 was utilized alongside the “xgb.train­()” function from the xgboost package to train an XGBoost regression model on the training set. Parameter adjustments were made by validating the results for the test set using the MSE criterion. The objective parameter “multi:softprob” in xgb.train­() is utilized in the classification task.

## Results and Discussion

### Extraction of True Virus SERS Spectra

Figure 2A shows the MMSs of HMPV-A with concentrations *c* ∈ *C* = {0, 50, ···, 100,000 PFU/m}. At *c* = 50 PFU/mL, the spectrum exhibits peaks at wavenumbers Δν = 489, 1394, and 1608 cm^–1^, which are attributed to environmental contamination in water. When *c* increases from 50 to 781 PFU/mL, these features still dominate the MMSs, although a new peak at Δν ≈ 659 cm^–1^ begins to emerge. When *c* reaches 6250 PFU/mL and higher, the spectral features at Δν ≈ 659 and 1608 cm^–1^ gradually dominate the entire spectrum. At *c* = 50,000 and 100,000 PFU/mL, the spectrum is characterized by strong features at Δν ≈ 659 cm^–1^, which significantly differ from the spectrum at *c* = 0 PFU/mL. Based on these spectral feature changes, the spectrum at the highest concentration, *c* = 100,000 PFU/mL (or the highest concentration for other viruses), can be considered the best approximation of the TVS for HMPV-A (or other viruses). Since the MMS at the highest concentration contains the largest proportion of virus signal relative to the background, it can serve as a reference for the TVS. Thus, these TVSs will be compared to the ETVSs. Following the procedure proposed in the above section, the MSs with concentrations ranging from 50 to 50,000 PFU/mL are used for the NN model, and the ETVS and *â*(*c*) specific to that virus type can be obtained.

Figure 2B–D compares the ETVS (red) with the reference TVS (i.e., spectrum at the highest concentration, 100,000 PFU/mL, black) for three viruses: HMPV-A, RSV-B1, and H1N1. Visually, the ETVSs closely match TVSs, showing only minor differences. The Pearson correlation coefficients (PCCs) between the ETVS and TVS for these viruses are 0.995, 0.979, and 0.939, respectively, indicating a strong correlation. Similarly, the root-mean-square errors (RMSEs) between ETVS and TVS are 0.066, 0.003, and 0.004, respectively, reflecting very small deviations. Additional data provided in Tables S2 and S3 of SI further summarize the PCCs and RMSEs for all 12 viruses. The PCC values range from 0.903 (for CoV-NL63) to 0.996 (for HMPV-B), while the RMSE values range from less than 0.001 (for Flu B, Ad5, and H3N2) to 0.066 (for HMPV-A). The near-unity PCC values and minimal RMSEs confirm the accuracy and reliability of the TVS extraction process. The small discrepancies between ETVS and TVS can be attributed to two factors: (1) The approximated reference TVS may not represent the true TVS, as they can be influenced by experimental conditions and measurement errors; (2) the ETVS is derived from MMS, which may introduce slight variations due to inherent measurement errors.

The concentration coefficients, *â*(*c*), obtained from the NN model along with the ETVS, represent a quantitative relationship between the spectral variations and virus concentration. Figure 2E plots *â*(*c*) versus *c* for HMPV-A, RSV-B1, and H1N1, respectively. The data reveal a clear positive correlation with *â*(*c*) increasing as *c* grows. Based on *â*(*c*)–*c* trend, a logistic function is used to fit the data,

5 $$â\left(c^{\prime },{c^{\prime }}_{0},k\right)=\frac{1}{1+e^{-k\left(c^{\prime }-{c\prime }_{0}\right)}}$$

where *c*′ = log­(*c*), *c*′_0_ is the midpoint parameter, and *k* is the scale parameter. The fitting results, represented by solid curves in Figure 2E, demonstrate that the logistic function accurately describes the relationship for each virus type. This implies that *â*(*c*) can effectively quantify the virus concentration once extracted from the NN model. For other viruses, the *â*(*c*)–*c* relationship can similarly be modeled by using the logistic function. The corresponding fitting parameters of each virus are summarized in Table S4 of SI, providing further validation of the model’s applicability across different virus types.

Once the ETVS are extracted, the predicted MMS (PMMS) *Î*_M_(*c*, Δν) at different concentrations *c* can be written as

6 $${Î}_{M}\left(c,\Delta \nu \right)=â\left(c\right){Î}_{T}\left(\Delta \nu \right)+\left(1-â\left(c\right)\right)I_{B}\left(\Delta \nu \right)$$

Figure 3A–F compares the PMMS and corresponding MMS at *c* = 781 and 25,000 PFU/mL for HMPV-A, RSV-B1, and H1N1, respectively. Visually, each PMMS matches well with the corresponding MMS. The PCC values for HMPV-A, RSV-B1, and H1N1 at *c* = 781 PFU/mL are 0.98, 1.00, and 1.00, respectively, while at *c* = 25,000 PFU/mL, they are 0.98, 1.00, and 1.00, respectively. All PCC values are extremely close to 1, indicating excellent prediction accuracy. Figure 3G summarizes the PCC values of average PMMS and MMS at different concentrations for all 12 viruses, showing that all PCC values exceed 0.9, with an average PCC of 0.979. This confirms the ability of the proposed procedure to accurately extract TVS from the spectra of the mixtures and validates the use of a linear combination of ETVS and BMS using *â*(*c*) for spectral augmentation.

**Figure 3:**
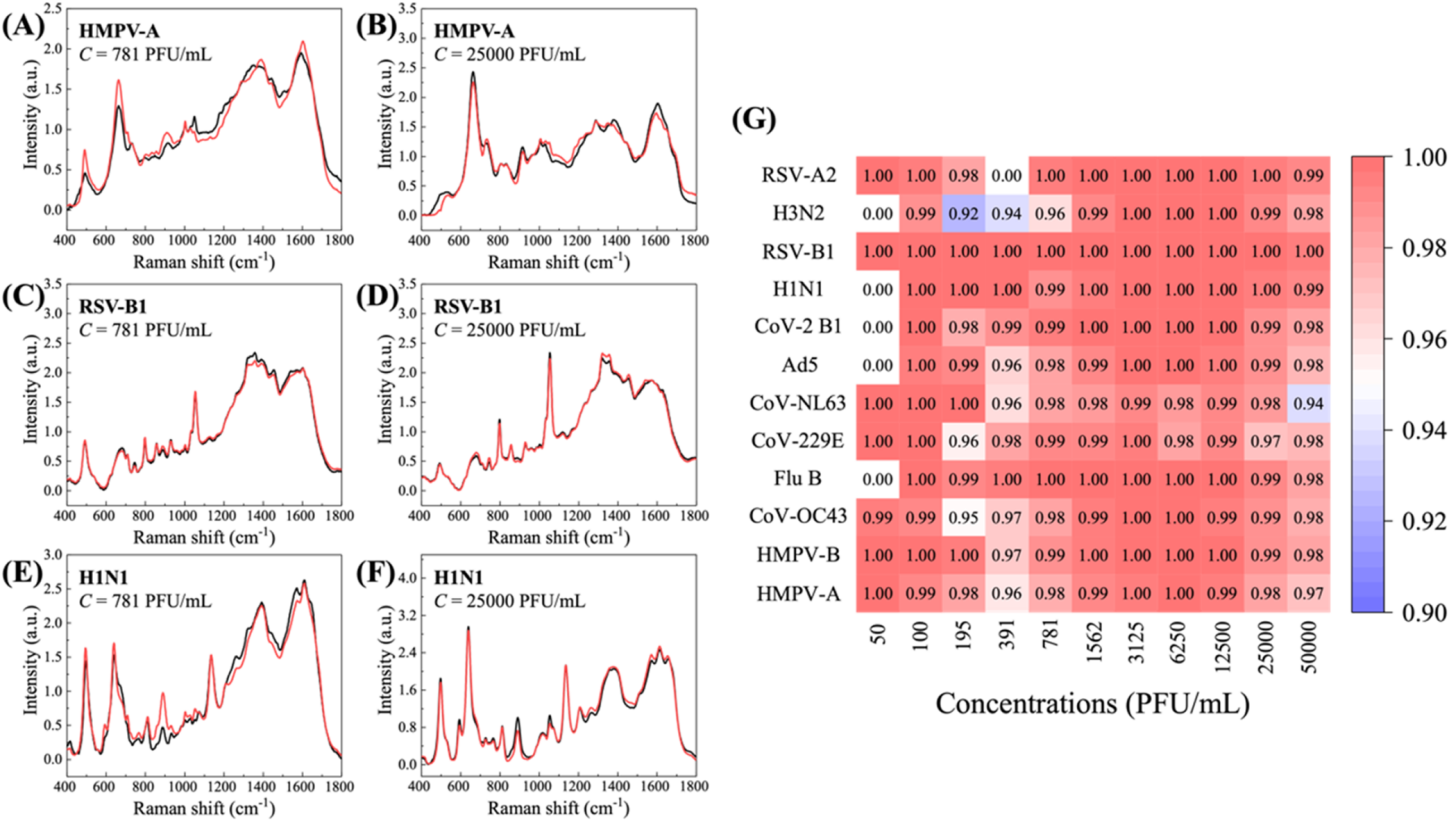
Comparison of the PMMS with the corresponding MMS at *c* = 781 and 25,000 PFU/mL for (A, B) HMPV-A, (C, D) RSV-B1, and (E, F) H1N1, respectively. Red curves are PMMS, and black curves are MMS. (G) The PCC heat map between PMMS and MMS among all viruses and concentrations.

The results in Figures 2 and 3 are based on the ETVS obtained from MMS of 10 different virus concentrations, covering both low- and high-concentration spectra. However, in practice, spectra from such a wide range of concentrations may not always be available, and only a limited number of concentrations might be accessible. To assess the performance of the proposed method under such constraints, a systematic study was conducted using combinations of 2–10 virus concentrations from the available data. For each virus, various concentration combinations were analyzed to extract the ETVS and compare them with TVS. Figure S6 demonstrates this for HMPV-B, showing ETVS extracted using two concentrations (*c* = 3125 and 6250 PFU/mL), three concentrations (*c* = 3125, 6250, and 12,500 PFU/mL), and four concentrations (*c* = 3125, 6250, 12,500, and 25,000 PFU/mL). The PCC values between ETVS and TVS are found to range from 0.940 to 0.972 for all combinations, indicating strong dependence on the concentration combinations and the number of concentrations used. A systematic evaluation of a two-concentration combination for HMPV-B (Table S5 of SI) reveals that the PCC values remain high (>0.94) when at least one concentration is very high, such as *c* = 50,000 or 25,000 PFU/mL, regardless of the other concentration. However, when both concentrations are low (*c* < 781 PFU/mL), the PCC values consistently fall below 0.8. By setting a PCC threshold of 0.9 as a benchmark for reliable ETVS extraction, we can define the threshold concentration *c*_th_. When both concentrations exceed *c*_th_, the PCC values between ETVS and TVS are consistently larger than 0.9; when either concentration is below *c*_th_, the PCC often drops below 0.9. For example, for HMPV-B, in Table S5, *c*_th_ for a two-concentration combination is determined to be 3125 PFU/mL. This threshold analysis is extended to three-, four-, and up to nine-concentration combinations. Table S6 summarizes the *c*_th_ values for all 12 viruses at different concentration combinations. A general trend emerges: as the number of concentrations used for ETVS extraction decreases, the *c*_th_ value increases. This trend reflects the greater reliance on individual concentration spectra when fewer concentrations are available. Higher *c*_th_ values correspond to higher signal-to-noise and signal-to-background ratios in the MMS, which are critical for the accurate ETVS extraction. Since the mixture contains both background and true virus information, a very low virus concentration results in a weak signal that can be overshadowed by the background. In such cases, the mixture spectra are dominated by background noise, making it difficult to isolate the true virus spectra. This issue is analogous to low signal-to-noise and signal-to-background ratio problems—when noise or background dominates the signal, extracting meaningful information becomes challenging.

The extracted true virus spectrum (ETVS) and true virus spectrum (TVS) are dependent on the SERS substrate (e.g., material and structure). In principle, SERS spectra can exhibit substrate dependence due to variations in surface plasmon resonance conditions, hot spot distributions, and molecular adsorption characteristics. However, in our study, we have taken specific steps to ensure that the extracted ETVS and TVS are minimally affected by substrate variations: (1) Consistent substrate usage: All experimental measurements were conducted using the same type of SERS substrate (AgNR@SiO_2_ array substrate). This minimizes substrate-induced spectral variations. (2) Spectrum extraction method: The neural network-based decomposition approach used to extract the ETVS relies on a linear decomposition model that separates virus-specific spectral features from background contributions. Since the extracted spectrum represents the intrinsic vibrational modes of the virus, it should be largely independent of minor substrate variations, provided that the virus is effectively adsorbed onto the SERS-active regions. (3) Plasmonic effects vs intrinsic spectral features: While SERS enhancement mechanisms can influence spectral intensity, shifts in peak position due to different substrates are typically small compared to the characteristic vibrational modes of the virus. Our extracted spectra demonstrate strong correlation (Pearson correlation coefficients >0.9) with those obtained at high concentrations, reinforcing that the dominant spectral features arise from viral molecular components rather than substrate-induced effects.

### Data Augmentation and Downstream Analysis

After obtaining the ETVS and PMMS for each concentration (ranging from 50 to 50,000 PFU/mL), the data augmentation process is carried out as described previously. For each virus, residuals are calculated as the difference between MS and MMS for the same concentration. To create synthetic spectra, these residuals are shuffled and added back to the PMMS, generating new augmented spectra for each concentration. This process is repeated multiple times to produce approximately 6000 synthetic spectra per virus, covering 12 concentrations (from 50 to 50,000 PFU/mL, about 500 spectra per concentration) for each virus. This results in the number of augmented spectra matching the number of measured spectra for each virus. Table S7 provides a summary of the measured and augmented spectral counts for all viruses. Once the data sets are augmented, they are randomly partitioned into training (80%) and validation (20%) subsets. After optimizing the XGBoost model using these subsets, all real measured MS from different viruses and concentrations are used as test spectra, enabling evaluation of the model’s performance.

An XGBoost classification model is developed using the augmented virus spectra data set for all 12 viruses. Each augmented spectrum is labeled with its true virus type and concentration. After training and validation, the optimized XGBoost model was then applied to classify virus types using experimental MS. The testing confusion matrix, shown in Figure 4A, demonstrates high accuracy with an overall classification accuracy of 0.923. Most viruses achieve an accuracy above 0.9, with the lowest being 0.803 for Flu B and the highest being 0.997 for RSV-A2. Additionally, the classification accuracy for each concentration of the 12 virus types is provided in Table S10 of SI. Classification accuracy generally improves with increasing concentrations, achieving near-perfect accuracy (∼1) for most viruses at 1000 PFU/mL and above. Some viruses, such as H1N1, RSV-B1, and RSV-A2, perform well even at lower concentrations, while others, such as HMPV-A, HMPV-B, Flu B, Ad5, and SARS-CoV-2 B, exhibit lower accuracy at low viral concentrations, reflecting challenges in distinguishing their spectral features.

**Figure 4:**
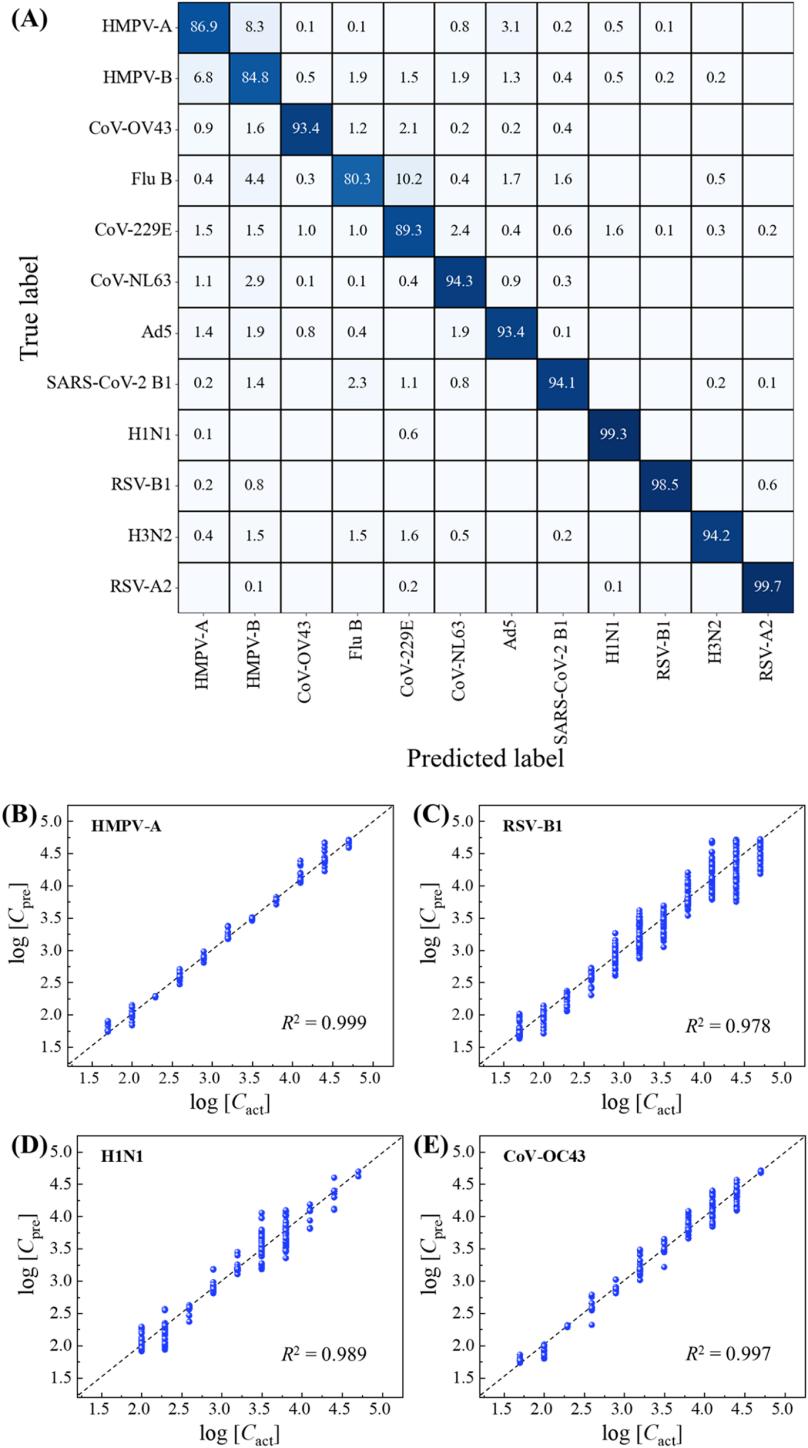
Results of classification and regression based on the XGBoost models using the augmented spectra for virus spectra in water: (A) The testing confusion matrix was used for classification. Comparison between the predicted concentrations *c*_pre_ resulted in the regression XGBoost model and the actual concentrations *c*_act_ for (B) HMPV-A, (C) RSV-B1, (D) H1N1, and (E) CoV-OC43. The black dashed lines represent log­(*c*_pre_) = log­(*c*_act_) (perfect prediction), and the blue data points represent the actual predicted concentrations.

Furthermore, Table S11 of SI highlights the model’s sensitivity, with an average value of 0.922. The highest sensitivity is 0.997 for RSV-A2, while the lowest is 0.791 for Flu B, attributed to spectral similarities between Flu B and HMPV-A, HMPV-B, and CoV-229E at low to mid concentrations, as shown in Figure S2. This similarity can lead to classification overlaps among these three viruses, resulting in a lower sensitivity for each, as shown in Figure 4A. Excluding these, all viruses achieve sensitivity above 0.9, demonstrating the model’s robustness in detecting virus presence.

To predict viral concentrations, a separate XGBoost regression model was trained for each virus using the augmented spectra from concentrations ranging from 50 to 50,000 PFU/mL. Figure 4B–E shows the plots of predicted concentrations log­(*c*_pre_) versus actual concentrations log­(*c*_act_) for HMPV-A, RSV-B1, H1N1, and CoV-OC43, respectively. Most predictions distribute closely along the ideal log­(*c*_pre_) = log­(*c*_act_) lines, as indicated by the dashed lines in the plots. For HMPV-A, H1N1, and CoV-OC43, as shown in Figure 4B,D,E, the predicted data points cluster well along this line, demonstrating a high prediction accuracy. Although the data for RSV-B1 are more scattered (Figure 4C), they still tend to group within a reasonable range around the log­(*c*_pre_) = log­(*c*_act_) line. The goodness of the concentration predictions is further quantified using the coefficient of determination, *R* ^2^ value, which measures how well the predicted data fit the line log­(*c*_pre_) = log­(*c*_act_). As shown in Figure 4B–E, the *R* ^2^ values are 0.999 for HMPV-A, 0.978 for RSV-B1, 0.989 for H1N1, and 0.997 for CoV-OC43, indicating highly accurate predictions. Similar results were obtained from other viruses, as summarized in Figure S7 and Table S12 of SI. Regardless of the virus type, all *R* ^2^ values exceed 0.95, confirming that the XGBoost models can effectively predict both virus concentration and virus type using the augmented spectra.

These results demonstrate that the augmented spectra can effectively emulate experimental MS, and the ETVS extracted by the NN model closely approximates TVS. The high classification accuracy and regression precision of the XGBoost models based on the augmented spectra validate their robustness in detecting and quantifying viruses in water, particularly at moderate to high concentrations.

The LODs for different viruses based on the augmentation method can be obtained as shown in Table S21 of SI, and the detailed calculation is described in Section S13. Overall, the LODs range from 16.9 to 62.2 PFU/mL, demonstrating relatively high sensitivity across different virus types. Among the viruses, SARS-CoV-2 B1 exhibited the lowest LOD (16.9 PFU/mL), indicating the highest detection sensitivity in the current setup. HMPV-A, HMPV-B, Ad5, H3N2, and RSV-A2 also showed low LODs (ranging from ∼23 to 27 PFU/mL), reflecting strong detection capabilities for these target viruses. In contrast, CoV-229E and CoV-NL63 displayed relatively higher LODs (62.2 and 53.0 PFU/mL, respectively), suggesting slightly reduced sensitivity for these particular viruses. Flu B and H1N1 also had moderate LODs (52.2 and 46.7 PFU/mL), indicating that while detection remains effective, further optimization may be needed for maximal sensitivity toward these influenza strains. Importantly, despite minor variations, the LODs for all viruses fall well within a practical detection range suitable for clinical applications, highlighting the robustness and broad applicability of the developed detection method.

While it is true that some prior reports have demonstrated detection of viruses or viral components at concentrations below 100 CFU/mL, many of these studies rely on SERS tags and additional amplification steps, which significantly increase complexity, cost, and preparation time. In contrast, the proposed method is label-free, requiring no surface functionalization, target-specific probes, or biochemical preprocessing. This design emphasizes simplicity, rapidity, and broad applicability, particularly for point-of-care or resource-limited settings. Furthermore, the broader diagnostic capability of the platform is noteworthy. As shown in Table S22, the method successfully classifies and quantifies 13 different respiratory viruses using a unified, label-free detection approach. To the best of our knowledge, this represents one of the most extensive demonstrations of direct SERS-based virus differentiation. In contrast, most prior SERS studies target only a limited number of viral species (typically 3–7) and often focus on either classification or quantification but not both simultaneously. To further enhance sensitivity while retaining the advantages of label-free detection, several future directions are proposed: (1) optimizing substrate morphology to increase hotspot density and uniformity, (2) employing simple physical or chemical methods to preconcentrate viral particles, and (3) integrating advanced data processing techniques, such as machine learning or deep learning algorithms, to extract weak but informative spectral features from complex data sets. These improvements could significantly enhance diagnostic sensitivity without compromising the operational simplicity of the platform.

### Validation Virus Classification and Quantification in Saliva

As discussed in the Introduction section, one of the purposes of TVS extraction is to use the ETVS for spectral augmentation for clinical samples. Here, based on the success of the above section, the ETVS and *â*(*c*) values obtained from the NN model extraction in water will be used to augment SERS spectra in a saliva background. The augmented MS spectrum is calculated based on the following equation:

7 $${Î}_{M}^{\mathrm{sal}}\left(c,\Delta \nu \right)=â\left(c\right){Î}_{T}\left(\Delta \nu \right)+\left(1-â\left(c\right)\right)I_{B}^{\mathrm{sal}}\left(\Delta \nu \right)$$

where *I*_B_^sal^ is the saliva BMS and *Î*_M_^sal^ is the PMMS in saliva. The accuracy of PMMS in saliva is verified in two ways. First, the average PMMS and measured MMS in saliva are directly compared. Figure S8A–F of SI shows PMMS and MMS plots at two different concentrations, *c* = 781 and 25,000 PFU/mL for HMPV-A, RSVB-1, and H1N1. For HMPV-A and RSV-B1, the PMMS closely aligns with the MMS at both concentrations, suggesting an accurate estimation of the ETVS and concentration coefficients. For H1N1, differences are observed at specific wavenumbers; both PMMS and MMS curves exhibit the same general trend, with similar patterns of increase, decrease, and peak locations. To further quantify the spectral similarity, the PCCs between PMMS and MMS are calculated, and a PCC heatmap across all concentrations and virus types is shown in Figure S8G. For all 12 viruses, the average PCC value is 0.954, with the lowest PCC value being 0.83, indicating a high degree of similarity between PMMS and MMS in saliva.

The accuracy of PMMS in saliva is further evaluated by using a data augmentation method similar to that applied in water. About 3800–3900 augmented spectra were generated for each virus at various concentrations, as summarized in Table S7. These augmented data sets are randomly divided into 80% for training and 20% for validation and optimization of the XGBoost models. After optimization, the experimentally measured SERS spectra of viruses in saliva are used for model testing. The testing confusion matrix (Figure 5A) for virus classification demonstrates high accuracy, with an overall accuracy of 0.919. Most viruses achieved accuracy values above 0.9, with the lowest accuracy being 0.838 for Ad5 and the highest accuracy being 0.990 for H3N2. Table S13 of SI highlights the XGBoost model’s classification accuracy across concentrations for 10 viruses in saliva. Similar to the results in water, accuracy improves with increasing concentrations, achieving perfect accuracy (∼1) for most viruses at 12,500 PFU/mL and above. Viruses like CoV-229E, CoV-NL63, RSV-B1, H1N1, and H3N2 perform consistently well across all concentrations (>0.85), while others, such as HMPV-A, CoV-OC43, Flu B, and Ad5, show lower accuracy at lower concentrations, with variability at intermediate concentrations. Table S14 of SI summarizes the sensitivity for each virus, which ranges from 0.838 (Ad5) to 0.990 (H3N2), with an average sensitivity of 0.919. Most viruses, such as H1N1 (0.976), RSV-B1 (0.980), and CoV-NL63 (0.975), demonstrate excellent sensitivity, indicating a strong model performance in detecting these viruses. Lower sensitivity for Ad5 (0.838) and CoV-OC43 (0.846) suggests that these viruses are slightly more challenging to classify.

**Figure 5:**
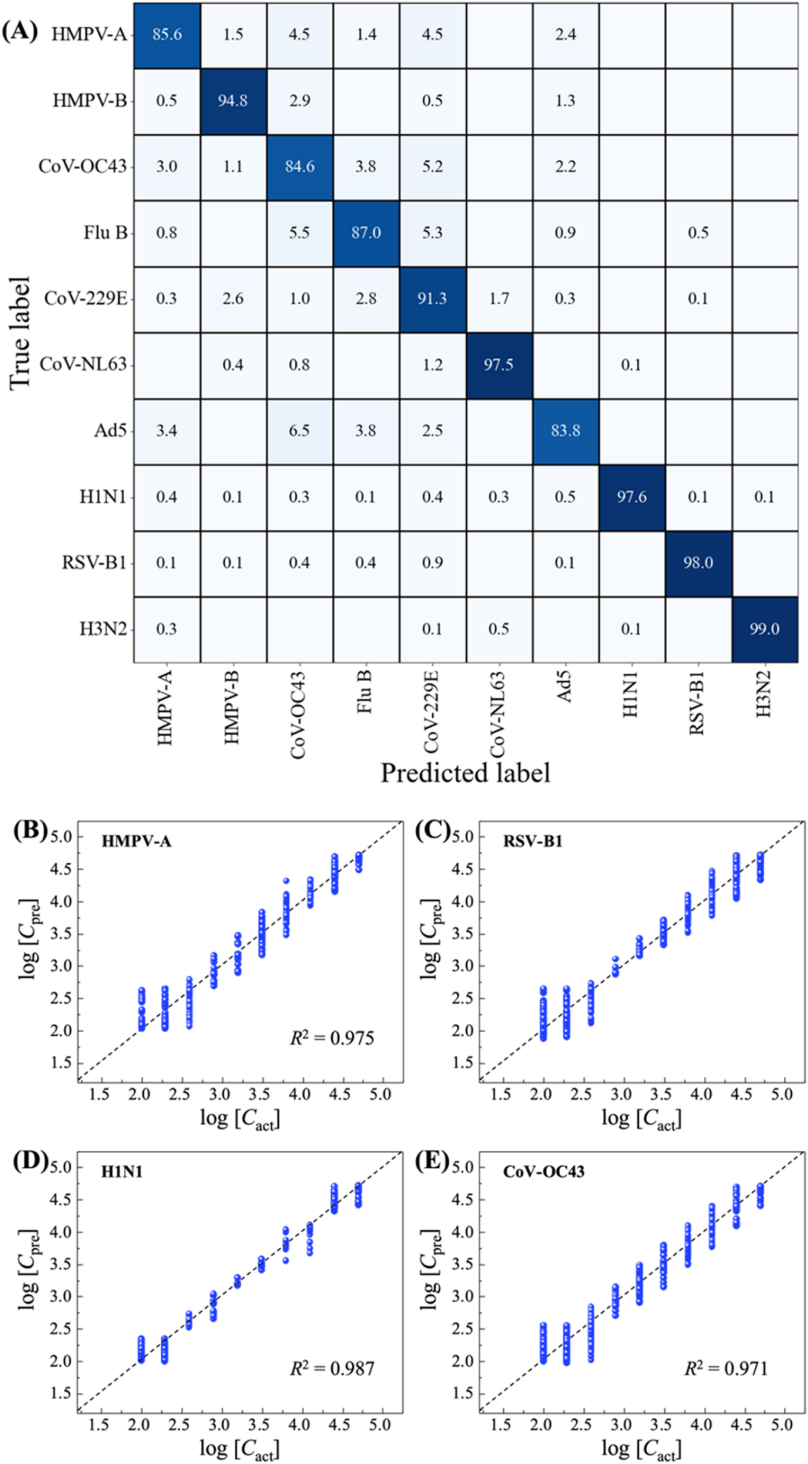
Results of classification and regression based on the XGBoost models using the augmented spectra for virus spectra in saliva: (A) The testing confusion matrix for classification. Comparison between the predicted concentrations *c*_pre_ resulted in the regression XGBoost model and actual concentrations *c*_act_ for (B) HMPV-A, (C) RSV-B1, (D) H1N1, and (E) CoV-OC43. The black dashed lines represent log­(*c*_pre_) = log­(*c*_act_) (perfect prediction), and the blue data points represent the actual predicted concentrations.

Similarly, XGBoost regression models are constructed for each virus using the augmented virus spectra in saliva. Figure 5B–E shows representative results for HMPV-A, RSV-B1, H1N1, and CoV-OC43. All of the predicted concentrations follow the log­(*c*_pre_) = log­(*c*_act_) line well, with *R* ^2^ values being 0.975, 0.987, 0.969, and 0.971, respectively. The predicted concentrations for other viruses are shown in Figure S9, and the corresponding *R* ^2^ values are summarized in Table S15 of SI. Overall, all of the *R* ^2^ values are larger than 0.93, which indicates excellent quantification capability of using augmented spectra to train the XGBoost regression models. These findings collectively suggest that the augmented data closely resemble the measured data. Furthermore, they demonstrate that the obtained PMMS in saliva captures the same features as the MMS. This, in turn, verifies that the ETVS obtained by our NN model extraction in a water buffer can be validated even when the background is saliva.

Deidentified human saliva specimens were collected from healthy, asymptomatic donors with no recent history of respiratory infections and confirmed to be virus-free. Viral contamination was assessed using reverse transcription polymerase chain reaction (RT-PCR) assays, which detected no viral RNA. In general, human saliva is a complex biological fluid composed primarily of water (∼99%), playing key roles in digestion, lubrication, antimicrobial defense, and oral homeostasis.^35–37^ It contains essential inorganic ions that regulate pH and mineral balance as well as key proteins and enzymes such as amylase (for starch digestion), lingual lipase (for lipid breakdown), mucins (for lubrication), and antimicrobial agents like lysozyme, lactoferrin, and immunoglobulin A (IgA). Metabolic byproducts, including urea, ammonia, and uric acid, reflect systemic health, while hormones and growth factors influence physiological processes. Additionally, saliva harbors a diverse microbiome, which contributes to oral health. It also contains exogenous substances from the diet, medications, and environmental exposure. Regarding potential interference from proteins and enzymes, these biomolecules could compete with virus particles for adsorption onto the SERS substrates, potentially affecting the signal reproducibility. By applying true virus spectrum extraction using a neural network-based decomposition approach, this method enables the isolation of virus-specific spectral features from background interference, reducing the impact of competitive adsorption effects. These findings further confirm that augmented saliva spectra based on ETVS closely replicate measured MP. The PMMS in saliva captures the same spectral features as the MMS, validating that ETVS extracted from the NN model in a water buffer remains accurate in a saliva background. The results demonstrate that the augmented data effectively enable robust virus classification and quantification, even in complex backgrounds like saliva.

## Conclusions

In this paper, a novel deep-learning-based strategy is proposed to extract true SERS spectra of viruses from measured spectra across different viral concentrations. The ETVS are free from noise and background interference, making them ideal for spectral augmentation and diagnostic applications in diverse environments. By using the virus spectra from the highest concentration in water as an approximation for the TVS, the method demonstrates that ETVS closely matches TVS, validating the robustness of the extraction approach. The method assumes that the MS can be represented as a linear combination of TVS and BMS, with the concentration coefficient *a*(*c*) accurately reflecting spectral changes with virus concentration. Using the ETVS, BMS in water, and predicted coefficient *â*(*c*), augmented virus SERS spectra are generated across various concentrations. This enables the development of deep learning models that achieve virus classification accuracy exceeding 92.3% regardless of concentration and concentration prediction with *R* ^2^ values consistently greater than 0.9. This strategy is further extended to saliva-based virus detection by combining the ETVS extracted from water with saliva-based BMS. The resulting classification and regression models achieve classification accuracy above 91.9% and concentration prediction with *R* ^2^ > 0.9 for all 10 viruses, even when tested on experimental virus-in-saliva spectra.

The ability to adapt ETVS for different biological backgrounds can ensure that the augmented spectra are representative of real-world conditions, enhancing the robustness and accuracy of DL models for virus detection and quantification. Thus, the proposed approach can offer the following advantages: (1) Efficient spectral augmentation: The method enables precise spectral augmentation using linear combinations of ETVS and BMS, eliminating the need for extensive experimental data collection across various backgrounds and concentrations. This allows DL models to train on diverse data sets without requiring new experiments for each condition. (2) Reduced experimental burden: By extracting TVS from limited measurements, such as high-concentration spectra in water, the method minimizes the need for labor-intensive and time-consuming experiments, significantly reducing the costs associated with reagents, substrates, samples, and equipment time. (3) Faster model development: Rapid data generation accelerates the training and validation of DL models, enabling faster iterations and improvements. This efficiency speeds up the development of diagnostic tools, reducing timelines from months to a fraction of the time.

This novel DL framework for SERS spectral extraction and enhancement has the potential for broad applications beyond viral detection. Future work could expand this methodology to other bioanalytes, such as bacteria, proteins, or small molecules, in various backgrounds, such as blood, urine, or food matrices. Additionally, this approach can be further refined for environmental monitoring, chemical detection, or industrial sensing, where background interference is common. The robustness and flexibility of this method suggest promising advancements in rapid diagnostics, point-of-care devices, and personalized medicine, where SERS-based detection systems could be paired with DL models for highly accurate real-time analysis. Future development could also focus on integrating this framework with portable SERS systems to create field-deployable diagnostic tools that are both cost-effective and scalable across different applications.

## Supplementary Material



## Notes

Detailed descriptions of model settings, results, and comparisons of our method (PDF)
