# Supplementary material for: Extracting True Virus SERS Spectra and Augmenting Data for Improved Virus Classification and Quantification
Source: ACS Sens. 2025 May 18;10(6):3941–52. doi: 10.1021/acssensors.4c03397 (PMC12210261; doi:10.1021/acssensors.4c03397)
Supplement: Supplementary file 1 [file se4c03397_si_001.pdf]

Supporting Information

Extracting True Virus SERS Spectra and Augmenting Data for Improved Virus Classification and Quantification

Yufang Liu [1#], Yanjun Yang [2#], Haoran Lu [1], Jiaheng Cui [3], Xianyan Chen [4], Ping Ma [1*], Wenxuan Zhong [1*] and Yiping Zhao [2*]

[1] Department of Statistics, Franklin College of Arts and Sciences, University of Georgia, Athens, Georgia 30602, United States

[2] Department of Physics and Astronomy, Franklin College of Arts and Sciences, University of Georgia, Athens, Georgia 30602, United States

[3] School of Electrical and Computer Engineering, College of Engineering, The University of Georgia, Athens, GA 30602, United States

[4] Department of Epidemiology & Biostatistics, College of Public Health, The University of Georgia, Athens, GA 30602, United States

[#] These authors contribute equally to this work

* Corresponding Authors: E-mail: pingma@uga.edu; wenxuan@uga.edu; zhaoy@uga.edu

S1. Virus incubation.

The following viruses were used in this study: SARS-CoV-2 (WA1/2020), SARS-CoV-2 B1.1.7 variant (SARS-CoV-2 B1), human coronavirus NL63 (CoV NL63), human coronavirus 229E (CoV 229E), human coronavirus OC43 (CoV OC43); influenza A H1N1 Brisbane (H1N1, IAV), influenza A H3N2 Hong Kong (H3N2, IAV), and influenza B (IBV); respiratory syncytial virus (RSV) from strain A2 (RSV-A2) and B1 (RSV-B1); human metapneumovirus (HMPV) from strain A (HMPV-A) and B (HMPV-B), as well as adenovirus type 5 (Ad5). These viruses are common respiratory viruses except for SARS-CoV-2 that is responsible for the COVID-19 pandemic. IAV, IBV, RSV-A2, RSV-B1, HMPV-A, and HMPV-B co-circulate in humans and may cause serious respiratory disease [1-3].

All viruses were propagated in Vero E6 cells which were maintained in Dulbecco's Modified Eagles Medium (DMEM; GIBCO BRL laboratories, Grand Island, NY) supplemented with 1% fetal bovine serum (FBS; Hyclone Laboratories, Salt Lake City, UT). Briefly, cells were infected using a multiplicity of infection (MOI) = 0.1. After 48 h, the viruses were harvested in serum-free DMEM followed by freeze-thaw after which the contents were collected and centrifuged at 4000 g for 15 min at 4°C. The virus titers were similar, i.e., 10^5 PFU/mL, as determined by plaque assay as previously described [4-6]. The reference specimen for these studies was diluted in DMEM supplemented with 1% FBS. Influenza strains, H1N1 and H3N2, were propagated in embryonated chicken eggs and virus titers determined by hemagglutination assay using chicken red blood cells. The influenza virus titers ranged between 10^7 -10^8 50% egg infectious dose (EID_{50}). The reference specimen for these studies was naive allantoic fluid. All the experiments on SARS-CoV-2 and SARS-CoV-2 variants were conducted in a biosafety level 3 (BSL-3) lab, while others were performed in a BSL-2 lab. All the experimental operations followed the biosafety guidelines: https://www.cdc.gov/coronavirus/2019-nCoV/lab/lab-biosafety-guidelines.html.

S2. Experimental materials.

Silver (Kurt J. Lesker, 99.99%) and titanium pellets (Kurt J. Lesker, 99.995%) were purchased as evaporation materials. Tetraethylorthosilicate (TEOS; Alfa Aesar, 99.9%), ammonium hydroxide (J. T. Baker, 28.0 - 30.0 wt.%) and ethanol (EtOH; Sigma-Aldrich, 95%) were used for silica shell growth. Dulbecco's Modified Eagles Medium (DMEM; GIBCO BRL

laboratories, Grand Island, NY) supplemented with 1% fetal bovine serum (FBS; Hyclone Laboratories, Salt Lake City, UT) was used as cell culture media. Pure water (Sigma-Aldrich) was used throughout all the experiments. All the reagents were used without further purification.

S3. Silver nanorod (AgNR) substrate fabrication.

Silver nanorod (AgNR) arrays prepared by the oblique angle deposition (OAD) are excellent SERS substrates as reported previously.[7-10] Briefly, clean glass slides (0.5 inch × 0.5 inch) were loaded into a vacuum deposition chamber with the substrate normal antiparallel to the incident vapor direction. A layer of 20 nm-thick Ti film (Titanium pellets, Kurt J. Lesker, 99.995%) and a layer of 200 nm-thick Ag film Silver (Silver pellets, Kurt J. Lesker, 99.99%) were deposited in sequence at a rate of 0.2 nm/s and 0.3 nm/s, respectively. Then, the substrate normal was rotated to 86° relative to the incident vapor direction, and a thickness of 2000 nm Ag film was deposited at a rate of 0.3 nm/s to obtain the AgNR arrays. The entire evaporation process was conducted under a high vacuum condition with a pressure < 3×10^{-6} Torr. According to previous extensive studies, the AgNR substrates have been demonstrated to possess good SERS reproducibility with <10% relative standard deviation (RSD), high SERS enhancement factor up to 10^9, and large area uniformity.[7, 8, 11, 12]

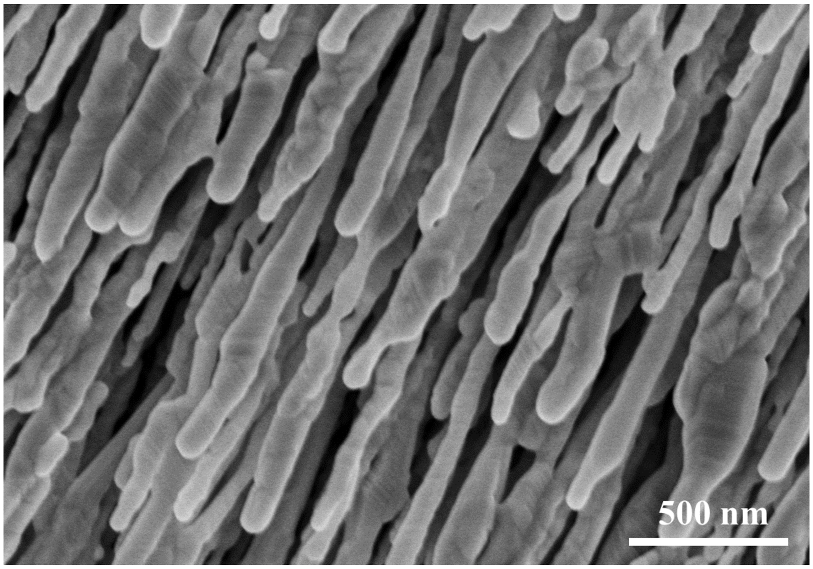

Figure S1 SEM image of AgNR@SiO_2 array SERS substrate.

To prevent the chemical degradation of AgNR in buffer solution, a very thin layer (~ 2 nm) of dense silica shell was uniform coated on the AgNRs. The silica shell growth conditions with

desired thickness were previously reported [13]. In this study, AgNR arrays were immerged into a homogeneous mixture composed of 30 mL of EtOH, 4 mL of H_2O, and 500 μL of TEOS for 20 min under stirring. Once 560 μL of ammonium hydroxide was added into the mixture, the reaction was initiated. The shell thickness was controlled by the hydrolysis time of TEOS after the accession of alkaline. The AgNR arrays were removed from the reaction solution after 5 min, followed by water rinsing and N_2 drying. Subsequently, a PDMS layer with arrayed small wells (4 wells, with the well diameter of 4 mm, well depth of 1 mm) was molded on the AgNR-SiO_2 array to restrict the effective sensing areas [14], referred to as AgNR-SiO_2 wells. A typical SEM image of an AgNR@SiO_2 array substrate is shown in **Figure S1**.

S4. Virus specimen preparation.

To obtain the SERS signal of the virus specimens at different concentrations, the virus was diluted with pure water. 5 μL of the diluted virus specimen was dispensed onto the AgNR-SiO_2 wells and air-dried at 20℃. Virus-inoculated saliva specimens were prepared by adding known (predetermined) concentrations (PFU) of virus specimens to achieve final concentrations ranging from 195 to 10^5 PFU/mL for SERS measurement.

S5. SERS characterization and data pre-processing.

The SERS spectra were collected from multiple randomly selected locations by using a Tec5USA Raman spectrometer (Tec5USA Inc.), with a 785 nm excitation wavelength and a ~100 μm (diameter) laser spot. The laser power was 32 mW and the acquisition time was 2 s. Based on the overall spectral features of SERS spectra obtained, a simple baseline correction method called "Gaussian-Lorentzian function fitting (GLFF)" was applied to obtain more uniform SERS spectra [15]. Then, area normalization is applied for each spectrum for further data analysis.

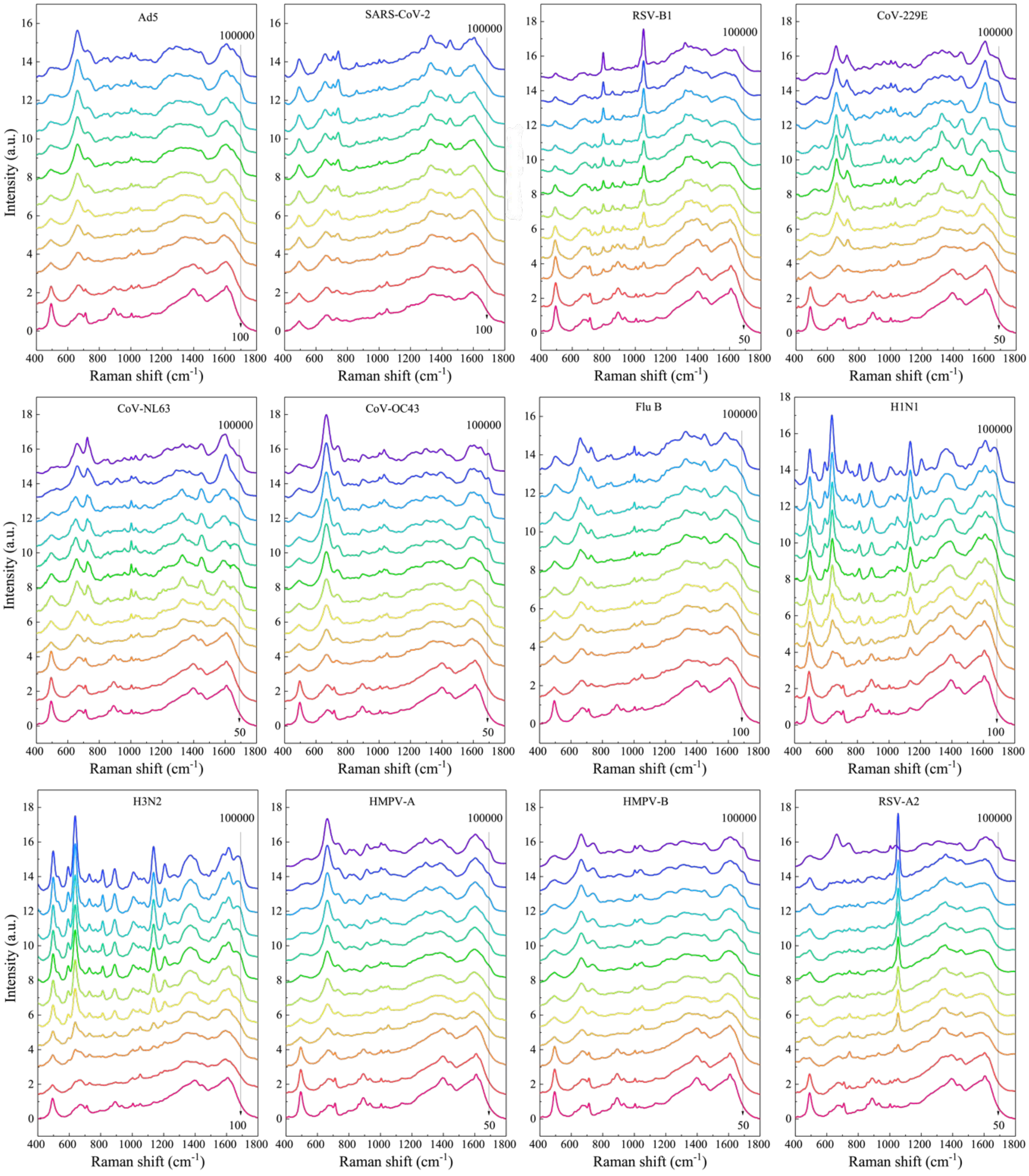

Figure S2 Concentration dependent SERS spectra plots for different viruses.

S6. Example for non-Gaussian, non-independent noise in measured spectra

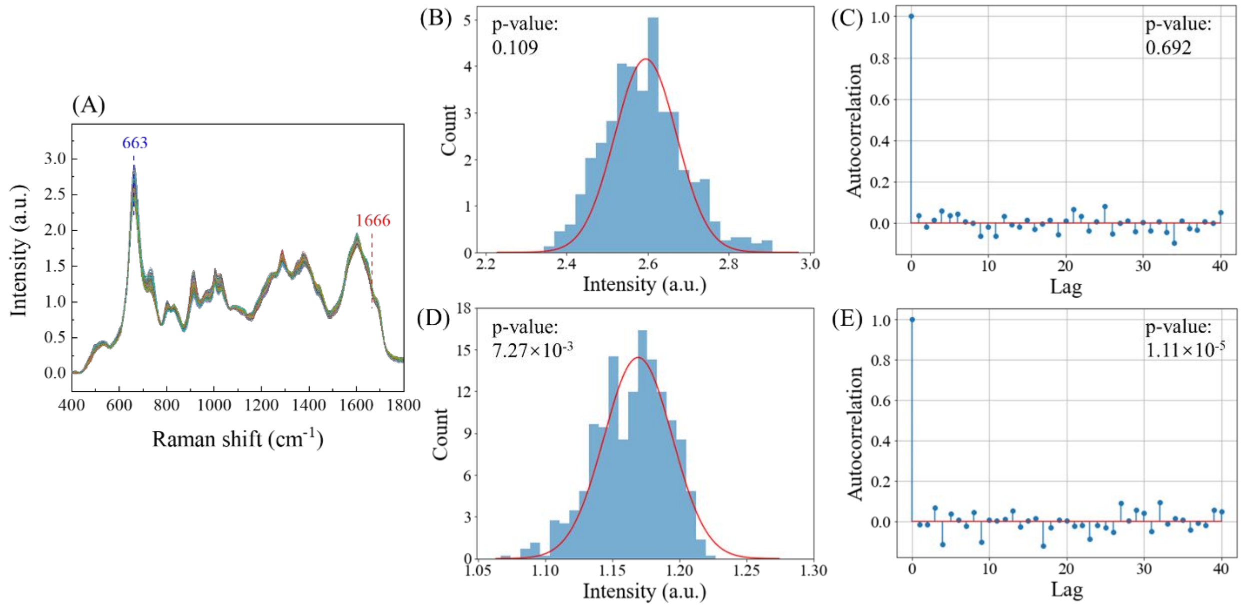

Figure S3. Analysis of normalized SERS spectra of HMPV-A at 50,000 PFU/mL: (A) 576 normalized spectra. (B) Intensity histogram and (C) autocorrelation function for 663 cm^{-1}. (D) Intensity histogram and (E) autocorrelation function for 1666 cm^{-1}.

The statistic variation and measurement correlation of the SERS virus spectra are illustrated using the normalized HMPV-A spectra at $c =$ 50,000 PFU/mL, with a total of 576 measurements, as shown in **Figure S3A**. To validate the statistical properties of the spectral intensity distribution at each wavenumber, histograms of intensity values at each wavenumber were generated for the spectral range of 400 to 1799 cm^{-1}. Shapiro-Wilk tests[16] were performed to assess the normality of these intensity distributions. In addition, the autocorrelation function $R(\tau, \Delta\nu)$ of the intensity values was computed to quantify the correlation between data points separated by a specific lag τ.[17] The autocorrelation function is defined by

$$R(\tau, \Delta\nu) = \frac{1}{N-\tau}\sum_{i=1}^{N-\tau}\left(I_i(\Delta\nu) - \bar{I}(\Delta\nu)\right)\left(I_{i+\tau}(\Delta\nu) - \bar{I}(\Delta\nu)\right), \quad \text{(S1)}$$

where $I_i(\Delta\nu)$ and $I_{i+\tau}(\Delta\nu)$ are the intensity of the i-th and $(i+\tau)$-th measurements, and $\bar{I}(\Delta\nu)$ is the average intensity at $\Delta\nu$. The Ljung-Box test[18, 19] was carried out to assess the time-independence of the spectral intensities at each wavenumber. A significance level, $\alpha = 0.05$, is set for both tests. For the Shapiro-Wilk tests, $p > \alpha$ signifies that we fail to reject the null hypothesis that the intensity distribution follows a Gaussian distribution; while for Ljung-Box tests, $p > \alpha$ indicated that we fail to reject the null hypothesis that the spectral intensity measurements

is time-independent.

Figures S3B and **C** show the intensity histogram and autocorrelation function at a fixed wavenumber $\Delta\nu = 663$ cm^{-1}. The histogram (**Figure S3B**) can be fit closely by a Gaussian distribution, as shown by the red curve, with the Shapiro-Wilk test yielding a p-value of 0.109 ($p > \alpha$), confirming the normality of the intensity distribution. The autocorrelation function R $(\tau, \Delta\nu)$ (**Figure S3C**) exhibits a sharp decay, with $R(\tau > 0, \Delta\nu)$ significantly smaller than R $(\tau = 0, \Delta\nu)$, implying a δ-function-like profile, indicating minimal correlation between adjacent data points. The Ljung-Box test further supports this observation with a p-value of 0.692, well above the 0.05 significance level, suggesting time-independence.

In contrast, **Figures S3D** and **E** show the intensity histogram and autocorrelation function at $\Delta\nu =$ 1666 cm^{-1}. The histogram (**Figure S3D**) is left-skewed, deviating from a Gaussian distribution, as confirmed by a Shapiro-Wilk test p-value of 7.27×10^{-3}, which is below the 0.05 significance level. The autocorrelation function $R(\tau, \Delta\nu)$ (**Figure S3E**) reveals significant negative correlation values at various τ, suggesting time-dependence. This observation is supported by the Ljung-Box test with a p-value of 1.11×10^{-5}, well below the 0.05 threshold.

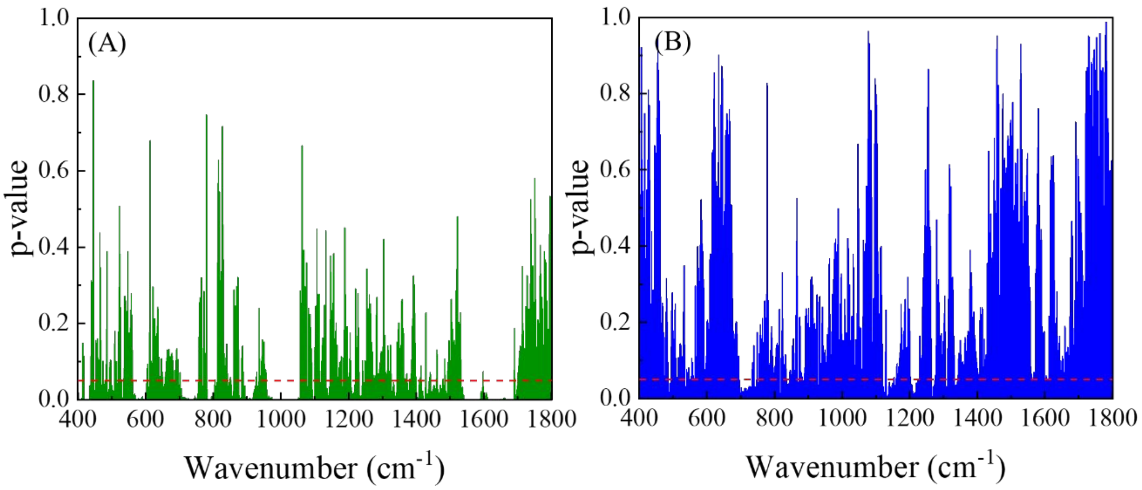

Figure S4 The p-values from (A) the Shapiro-Wilk and (B) Ljung-Box tests for the full wavenumber range for HMPV-A at 50,000 PFU/mL. The red dashed line represents the significance level (0.05)

Figure S4A plots the p-values from the Shapiro-Wilk tests, and **Figure S4B** presents the p-values from the Ljung-Box tests for the spectral range 400 to 1799 cm^{-1} for HMPV-A at $c =$ 50,000 PFU/mL. The red dashed lines in both figures represent the significance level ($\alpha = 0.05$).

Both **Figures S4A** and **B** reveal that a substantial portion of wavenumber regions have $p < \alpha$, indicating the presence of non-Gaussian and time-dependent spectra in the measurements. Based on these observations, the percentage of wavenumber regions with $p > \alpha$ and $p < \alpha$ was estimated. **Table S1** provides a summary of these percentages for all 12 viruses at $c =$ 50,000 PFU/mL. The percentage of wavenumber regions exhibiting Gaussian distribution varies widely among the viruses, with HMPV-B showing the highest proportion (55.6%) and CoV2 the lowest (19.5%). This variation suggests significant differences in the statistical properties of spectral intensity distributions, potentially influenced by the orientation of viruses in SERS substrate hotspots and the statistical distributions of hotspots on the SERS substrates. The percentage of time-independent wavenumber regions also exhibits substantial variability, with Ad5 showing the highest value (92.1%), indicating highly stable spectral intensity measurements over time. In contrast, viruses like RSV-A2 and RSV-B1 exhibit remarkably low time-independent percentages (1.4% and 6.4%, respectively), pointing to significant temporal correlations in their spectral intensities. This small p-value may be due to the systematic similarity caused by the uniformity of the SERS substrate, and consistent sample preparation.

Table S1. Summary of the percentages of wavenumber regions exhibiting Gaussian distribution and time-independence for viruses at 50,000 PFU/mL.

Virus	Gaussian percentage	Time-independent percentage
Ad5	23.5	92.1
CoV2	19.5	56.6
CoV229E	47.9	90.4
CoV2B1	20.5	61.6
CoV NL63	31.7	2.4
CoV OC43	30.4	68.1
Flu B	29.0	41.2
H1N1	27.9	77.4
H3N2	50.6	83.1
HMPV-A	44.3	80.2
HMPV-B	55.6	66.1
RSV-A2	40.4	1.4
RSV-B1	43.1	6.4

S7. Neural network structure for spectrum extraction.

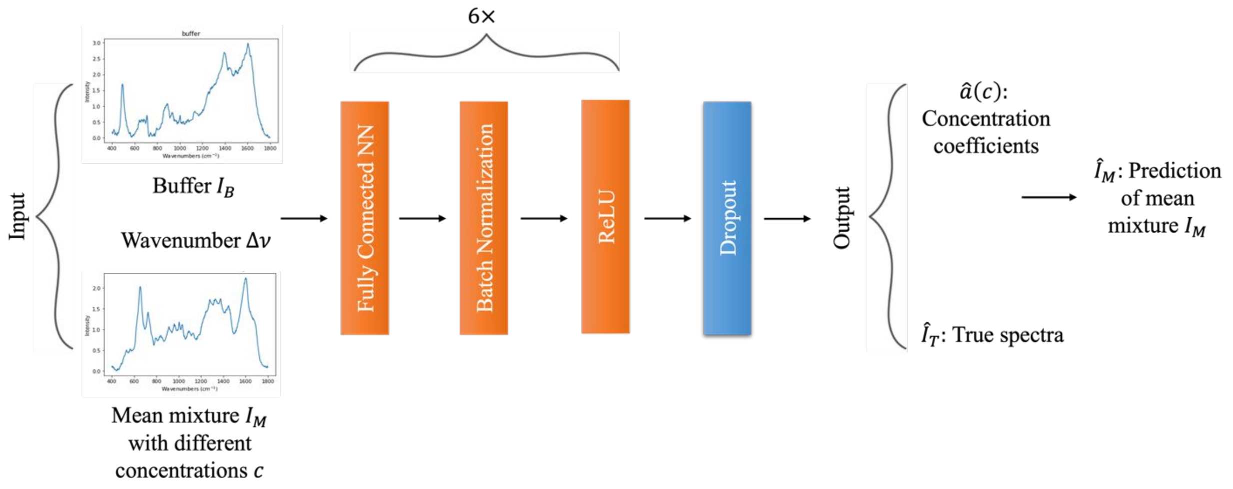

Figure S5 The structure of the neural network for spectrum extraction.

The structure of the NN model is shown in **Figure S5**. The inputs are intensities, wavenumbers $\Delta\nu$, concentrations c, BMS I_B and MMS I_M. The outputs are ETVS $\hat{I}_T$ and concentration coefficients $\hat{a}(c)$, as well as PMMS $\hat{I}_M$ by minimizing the loss function the equation: $L = \sum_{c\in C}\sum_{\Delta\nu=400}^{1799}\left(I_M(c,\Delta\nu) - \hat{a}(c)\hat{I}_T(\Delta\nu) - (1-\hat{a}(c))I_B(\Delta\nu)\right)^2$.The total number of measured spectrum is provided in **Table S8** and **Table S9** of **SI**. In a single virus extraction, there are 11 different concentrations, each with its own mean of measure spectrum (MMS), resulting in a total of 11 data points. Additionally, with background medium spectrum (BMS), the total number of data points for one virus extraction is 12.

S8. Extraction of true virus SERS spectra.

Table S2. Pearson correlation coefficients between ETVS and TVS.

HMPV-A	HMPV-B	CoV-OC43	Flu B	CoV-229E	CoV-NL63	Ad5	SARS-CoV-2 B1	H1N1	RSV-B1	H3N2	RSV-A2
0.995	0.996	0.962	0.984	0.981	0.903	0.964	0.969	0.939	0.979	0.978	0.931

Table S3. RMSEs between ETVS and TVS.

HMPV-A	HMPV-B	CoV-OC43	Flu B	CoV-229E	CoV-NL63	Ad5	SARS-CoV-2 B1	H1N1	RSV-B1	H3N2	RSV-A2
0.066	0.006	0.008	<0.001	0.050	0.006	<0.001	0.003	0.004	0.003	<0.001	0.024

Table S4. Obtained fitting parameters for logistic function fitting for 12 viruses.

Parameter / Virus	HMPV-A	HMPV-B	CoV-OC43	Flu B	CoV-229E	CoV-NL63	Ad5	SARS-CoV-2 B1	H1N1	RS-VB1	H3N2	RSV-A2
c_0	9.32	8.85	9.06	6.05	7.91	9.80	8.98	6.88	10.51	9.33	10.21	9.82
k	0.52	0.61	0.44	0.23	0.94	0.35	0.44	0.25	0.57	0.62	0.48	0.22

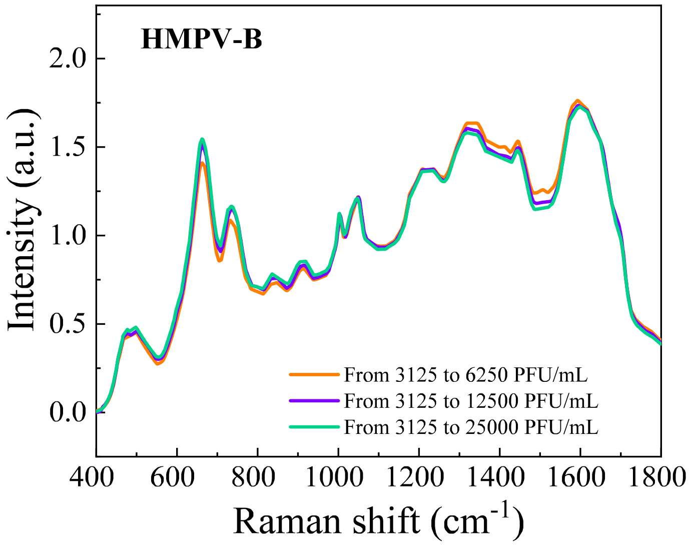

Figure S6 TVS extracted using different combinations of concentrations for HMPV-B: Orange: 2 concentration combination, c = 3125 and 6250 PFU/mL; Blue: 3 concentration combination, c = 3125, 6250 and 12500 PFU/mL; and Green: 4 concentration combination, c = 3125, 6250, 12500, and 25000 PFU/mL.

Table S5. PCC values of ETVS extracted using 2-concentration combination for HMPV-B.

**HMPV-B**	**195**	**391**	**781**	**1562**	**3125**	**6250**	**12500**	**25000**	**50000**
**100**	0.675	0.643	0.777	0.856	0.908	0.972	0.977	0.970	0.996
**195**	\	0.623	0.754	0.840	0.890	0.930	0.953	0.982	0.995
**391**		\	0.686	0.748	0.804	0.854	0.874	0.938	0.991
**781**			\	0.787	0.840	0.881	0.903	0.946	0.961
**1562**				\	0.871	0.924	0.938	0.959	0.980
**3125**					\	0.940	0.942	0.971	0.991
**6250**						\	0.976	0.987	0.993
**12500**							\	0.987	0.996
**25000**								\	0.996
**50000**									\

Table S6. The threshold of lowest viral concentration that can maintains a PCC value between ETVS and TVS greater than 0.9 for each virus at different numbers of concentration combination.

Virus/ Number of concentrations	2	3	4	5	6	7	8	9	10	11
**HMPV-A**	6250	3125	3125	1562	781	781	391	195	100	50
**HMPV-B**	3125	1562	1562	781	781	391	195	100	50	50
**CoV-OC43**	6250	3125	3125	1562	781	781	391	195	100	50
**Flu B**	1562	781	781	391	391	195	195	100	100	/
**CoV-229E**	3125	1562	781	391	391	195	100	50	50	50
**CoV-NL63**	12500	12500	6250	6250	3125	1562	781	391	100	50
**Ad5**	6250	3125	3125	1562	1562	781	391	195	100	/
**SARS-CoV-2 B1**	1562	1562	781	781	391	391	195	100	100	/
**H1N1**	12500	6250	6250	3125	1562	781	391	195	100	/
**RSV-B1**	1562	781	391	195	100	50	50	50	50	50
**H3N2**	3125	1562	1562	781	781	391	195	100	100	/
**RSV-A2**	3125	3125	1562	781	781	195	195	100	50	/

S9. Data augmentation and Downstream analysis.

Table S7. Number of augmented spectra in different backgrounds for different viruses.

Virus / Background	Water	Saliva
**HMPV-A**	6516	3969
**HMPV-B**	6459	3956
**CoV-OC43**	6477	3936
**Flu B**	5895	3933
**CoV-229E**	6424	3914
**CoV-NL63**	6289	3865
**Ad5**	5903	3954
**SARS-CoV-2 B1**	5892	\
**H1N1**	5926	3837
**RSV-B1**	6479	3963
**H3N2**	5889	3814
**RSV-A2**	5902	\

Table S8. Number of trained and tested samples in water backgrounds for different viruses.

Virus / Dataset	Train	Test
**HMPV-A**	5213	1303
**HMPV-B**	5239	1310
**CoV-OC43**	5182	1295
**Flu B**	4716	1179
**CoV-229E**	6424	1285
**CoV-NL63**	5031	1258
**Ad5**	4722	1181
**SARS-CoV-2 B1**	4714	1178
**H1N1**	4741	1185
**RSV-B1**	5183	1296
**H3N2**	4711	1178
**RSV-A2**	4722	1180

Table S9. Number of trained and tested samples in saliva backgrounds for different viruses.

Virus / Dataset	Train	Test
**HMPV-A**	3175	794
**HMPV-B**	3165	791
**CoV-OC43**	3149	787
**Flu B**	3146	787
**CoV-229E**	3131	783
**CoV-NL63**	3092	773
**Ad5**	3163	791
**H1N1**	3070	767
**RSV-B1**	3170	793
**H3N2**	3051	763

Table S10. Predicted classification accuracy for 12 viruses in water at different concentrations using XGBoost.

Virus/*c*	50	100	195	391	781	1562	3125	6250	12500	25000	50000
**HMPV-A**	0.436	0.290	0.829	0.992	0.992	1	1	1	1	1	1
**HMPV-B**	0.509	0.603	0.565	0.739	0.923	0.975	0.991	1	1	1	1
**CoV-OC43**	0.880	0.759	0.778	0.873	0.991	0.991	1	1	0.992	1	1
**Flu B**	/	0.603	0.636	0.672	0.669	0.669	0.846	0.857	0.966	0.966	0.899
**CoV-229E**	0.822	0.748	0.525	0.752	1	1	1	0.991	1	1	1
**CoV-NL63**	0.681	0.939	0.816	0.946	0.964	0.964	1	1	1	1	1
**Ad5**	/	0.470	0.983	0.882	1	1	1	1	0.992	1	1
**SARS-CoV-2 B1**	/	0.547	0.864	0.983	1	1	1	1	1	1	1
**H1N1**	/	0.975	0.966	1	1	1	1	1	1	1	0.992
**RSV-B1**	0.915	0.958	0.975	0.983	1	1	1	1	1	1	1

**H3N2**	/	0.578	0.840	0.992	1	1	1	1	1	1	1
**RSV-A2**	0.983	0.983	1	/	1	1	1	1	1	1	1

Table S11. Sensitivity of XGBoost classification for each virus.

HMPV-A	HMPV-B	CoV-OC43	Flu B	CoV-229E	CoV-NL63	Ad5	SARS-CoV-2 B1	H1N1	RSV-B1	H3N2	RSV-A2
0.869	0.847	0.934	0.791	0.893	0.940	0.934	0.940	0.993	0.985	0.941	0.997

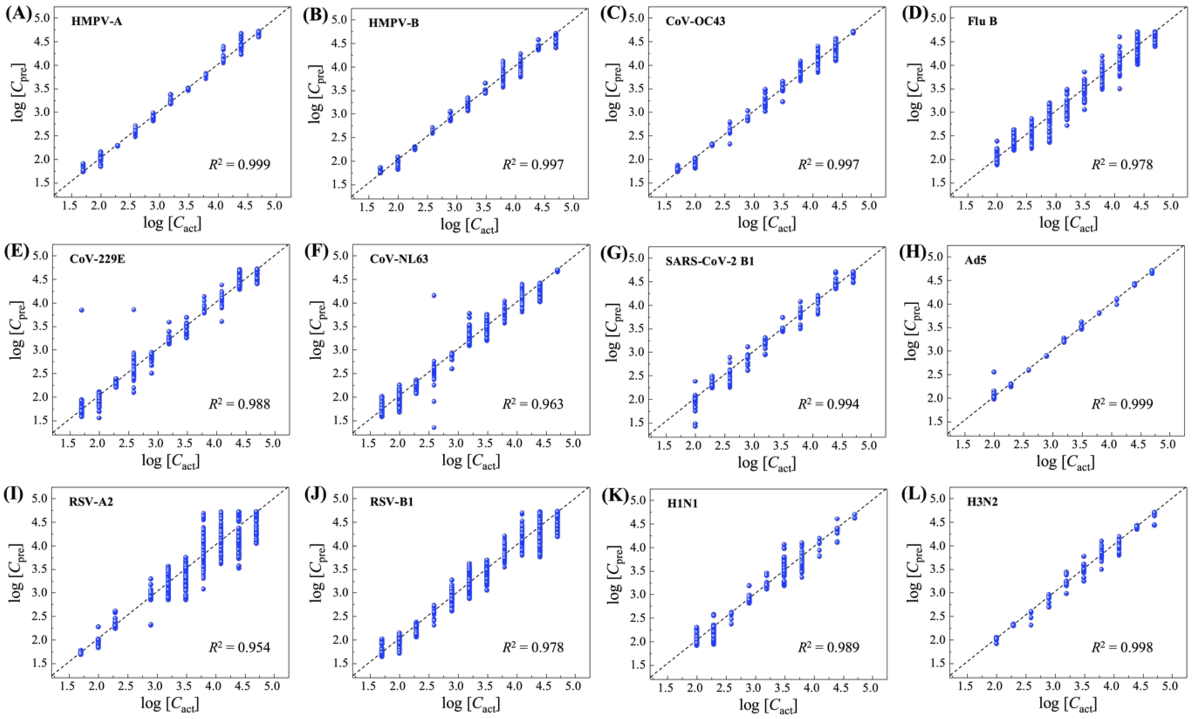

Figure S7. The XGBoost regression results for all viruses in water: (A) HMPV-A, (B) HMPV-B, (C) CoV-OC43, (D) Flu B, (E) CoV-229E, (F) CoV-NL63, (G) SARS-CoV-2 B1, (H) Ad5, (I) RSV-A2, (J) RSV-B1, (K) H1N1, (L) H3N2.

Table S12. The summary of R^2 value from XGBoost regression results in **Figure S7** for each virus in water.

Virus	HMPV-A	HMPV-B	CoV-OC43	Flu B	CoV-229E	CoV-NL63	Ad5	SARS-CoV-2 B1	H1N1	RSV-B1	H3N2	RSV-A2
	0.999	0.997	0.996	0.979	0.988	0.964	0.999	0.993	0.990	0.979	0.987	0.953

S10. Additional results for spectral augmentation for viruses in saliva.

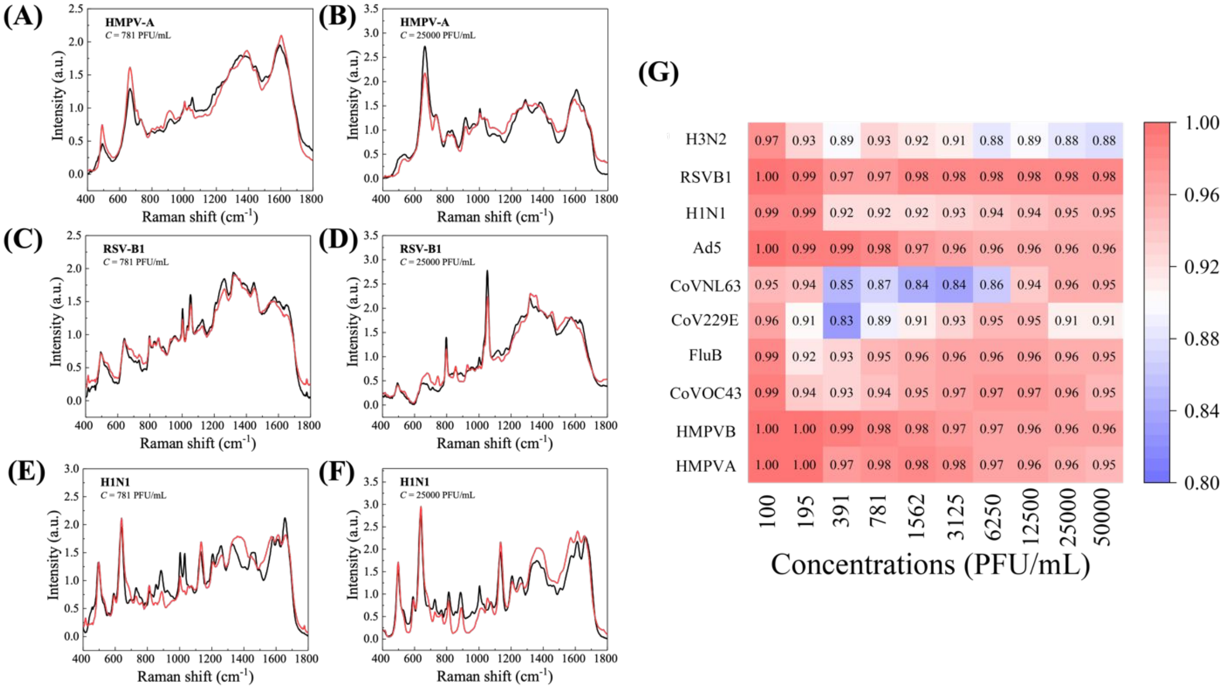

Figure S8. Comparison of the PMMS and corresponding MMS at and   PFU/ml for HMPV-A (A-B), RSV-B1 (C-D), and H1N1 (E-F), respectively, in saliva. (G) Correlation heat map between PMMS and MMS among all 10 viruses and different concentrations.

Table S13. Predicted classification accuracy for 10 viruses in saliva at different concentration using XGBoost.

**Virus/*c***	**100**	**195**	**391**	**781**	**1562**	**3125**	**6250**	**12500**	**25000**	**50000**
**HMPV-A**	0.526	0.684	0.628	0.825	0.950	0.975	0.962	1	1	1
**HMPV-B**	0.743	0.873	0.873	0.987	1	1	1	1	1	1
**CoV-OC43**	0.684	0.613	0.462	0.886	0.937	0.936	0.949	1	1	1
**Flu B**	0.633	0.625	0.705	0.848	0.949	0.987	0.987	1	1	0.974
**CoV-229E**	0.962	0.987	0.949	0.949	0.872	0.911	0.779	0.908	0.810	1
**CoV-NL63**	0.909	0.974	1	0.986	1	1	1	0.885	1	1
**Ad5**	0.304	0.342	0.759	0.975	1	1	1	1	1	1
**H1N1**	0.846	0.924	1	1	1	1	1	1	1	1
**RSV-B1**	0.886	0.937	0.974	1	1	1	1	1	1	1
**H3N2**	0.901	0.987	0.987	1	0.974	1	1	0.987	1	1

Table S14. Sensitivity of XGBoost classification for each virus in saliva.

**HMPV-A**	**HMPV-B**	**CoV-OC43**	**Flu B**	**CoV-229E**	**CoV-NL63**	**Ad5**	**H1N1**	**RSV-B1**	**H3N2**
0.856	0.948	0.846	0.870	0.913	0.975	0.838	0.976	0.980	0.990

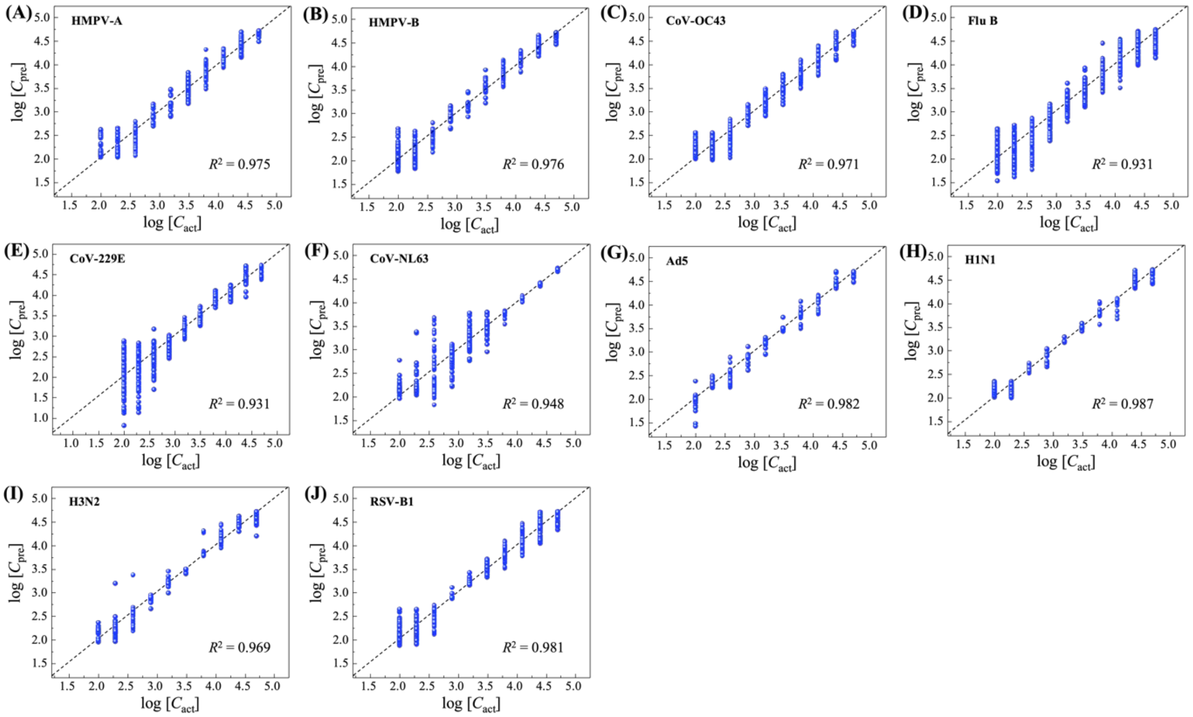

Figure S9. The XGBoost regression results for all viruses in saliva: (A) HMPV-A, (B) HMPV-B, (C) CoV-OC43, (D) Flu B, (E) CoV-229E, (F) CoV-NL63, (G) Ad5, (H) H1N1, (I) H3N2, (J) RSV-B1.

Table S15. The summary of R^2 value from XGBoost regression results in **Figure S9** for each virus in saliva.

Virus	HMPV-A	HMPV-B	CoVOC43	FluB	CoV229E	CoVNL63	Ad5	H1N1	RSVB1	H3N2
	0.975	0.976	0.971	0.931	0.931	0.948	0.982	0.987	0.969	0.981

To demonstrate the effectiveness of our augmentation approach in accurately capturing features within the measured mixture spectra, we conducted a comparative analysis with the augmentation techniques outlined in Ref. [20] and Ref. [21]. Following the methodologies described in these references, we initially horizontally shifted each spectrum randomly by a few wavenumbers, followed by the addition of random noise to each spectrum. Subsequently, we computed a linear combination of the resultant spectra with coefficients randomly set within the range from 0 to 1. This approach was then contrasted with our own data augmentation method applied in the context of multi-virus classification. The comparative results are presented in **Table S16**.

Table S16. Comparison of overall accuracy for multi-virus classification between our method and those reported in Ref. [20] and Ref. [21] for viruses in both water and saliva.

Background / Method	Our method	Method in Ref. [20] and Ref. [21]
Water	0.923	0.910
Saliva	0.919	0.904

Our findings demonstrate that using the proposed data augmentation method can achieve higher accuracy in virus classification for both water and saliva samples. This highlights the effectiveness of our augmentation technique in capturing the distinctive features within the measured mixture spectra.

Table S17. Comparison of overall accuracy for multi-virus classification among XGBoost model, SVM model and Random Forest model for water background.

Method / Criterion	Accuracy	Computational Time
XGBoost	**0.923**	44.86 sec
SVM	0.802	922.38 sec
Random Forest	0.916	**40.05 sec**

Python 3.9 was used for the analysis. The SVM model[22] was implemented using the 'SVC()' function from the 'sklearn.svm' package with a regularization parameter of C = 10, a Radial Basis Function (RBF) kernel, and a kernel coefficient set to 'scale'. The Random Forest model[23] was implemented using the 'RandomForestClassifier()' function from the 'sklearn.ensemble' package, with 100 decision trees and a maximum tree depth of 15. The training and testing samples were identical to those used in the XGBoost model analysis (As shown in **Table S8** & **S9** of **SI**). Model accuracy and computational time were evaluated on the test dataset. As shown in Table S17, the XGBoost model achieved the highest classification accuracy while maintaining comparable computational efficiency.

S11. Additional results for spectrum extraction.

Table S18. Pearson correlation coefficients between ETVS and TVS for CNN and NN methods in water background.

Method / PCC value	HMPV-A	HMPV-B	CoV-OC43	Flu B	CoV-229E	CoV-NL63	Ad5	SARS-CoV-2 B1	H1N1	RSV-B1	H3N2	RSV-A2
NN	0.995	0.996	0.962	0.984	0.981	0.903	0.964	0.969	0.939	0.979	0.978	0.931
CNNs	0.966	0.970	0.962	0.965	0.980	0.917	0.965	0.952	0.931	0.978	0.976	0.948

Table S19. Computational time between ETVS and TVS for CNN and NN methods.

Method	Computational Time
NN	**302.42 sec**
CNN	1994.50 sec

We have investigated the use of CNNs24 for ETVS. The CNN architecture consists of six convolutional layers with a kernel size of 3, each followed by a ReLU activation function and Batch Normalization. After pooling, two fully connected layers are included. The Pearson correlation coefficient (PCC) is computed between ETVS and TVS for both CNN and NN models, along with their respective computational times. As shown in Table S18, the PCC values for each virus are very similar between NN and CNN, indicating comparable feature extraction capabilities. However, Table S19 also shows that CNN requires approximately six times the computational cost for training compared to the NN model. This demonstrates that the NN model is more efficient, requiring less training time while still achieving high accuracy.

S12. Reproducibility of Raman spectra measurements.

Raman spectra can exhibit variations even when the same sample is measured multiple times. Therefore, it is crucial to verify whether the model is accurate and whether the measured spectra consistently demonstrate the same similarity to the Raman spectra of viruses. In the original SERS spectra (**Figure S10 (A)**), variations in intensity and fluctuations are observed across multiple measurements, primarily due to baseline variations and background noise. However, after baseline correction and average normalization, the SERS spectra (**Figure S10 (B)**) exhibit highly consistent peak locations and intensities across all measurements, indicating enhanced reproducibility.

As described in the manuscript, we use the mean of measured spectra (MMS) as input for extracting the true virus spectrum. It is true that experimental errors exist in the measured mixture spectra; this is why we take multiple measurements of the same spectrum and use their average as

input—to minimize the impact of experimental errors. When the number of measurements is sufficiently large, these errors become negligible.

To demonstrate this, we conducted an experiment showing how the experimental error can be negligible with our measurements. For each virus concentration, the mixture spectrum was measured approximately 600 times from several replicated SERS samples. In the manuscript, we take the average of these 600 measurements as input to directly extract the true virus spectrum. To further validate this approach, we take HMPV-A virus as an example and split the 600 measurements into two equal parts: the first 300 and the second 300. We then computed the average of each subset separately and extracted two virus spectra—one from each subset. These two spectra were then compared with the virus spectrum obtained using the full dataset as we have done in the manuscript. The results are shown in **Figure S11**.

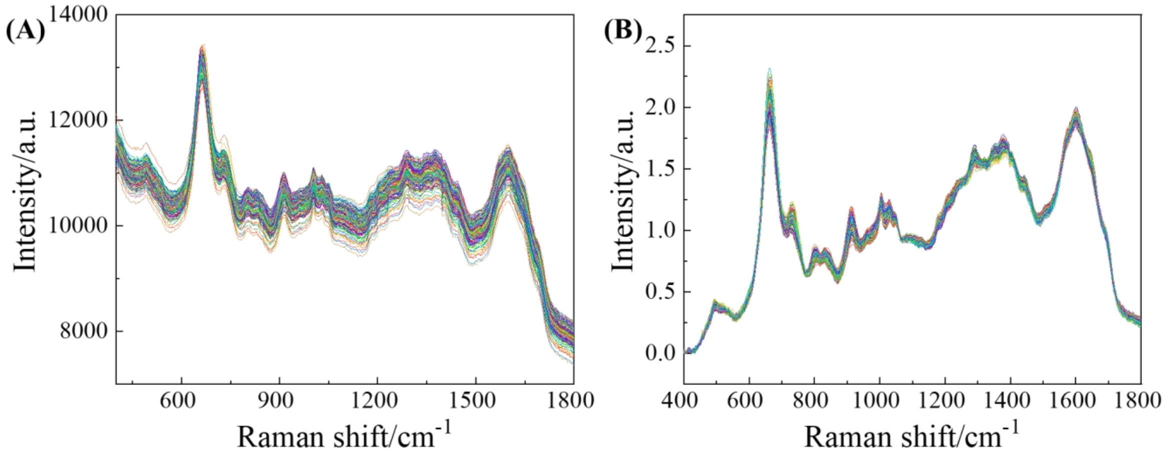

Figure S10. (A) Original SERS spectra of HMPV-A (6,250 PFU/mL). (B) Baseline removed and average-normalized the spectra.

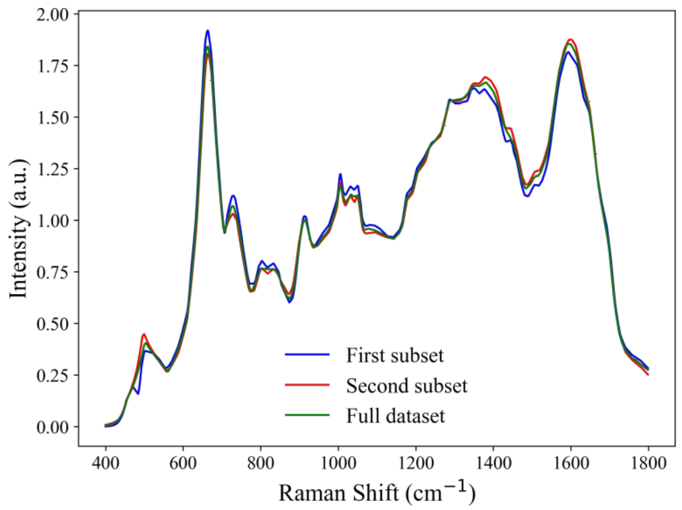

Figure S11: TVS extracted using different subdatsets as input for HMPV-A: Blue: subset of first 300 measured spectra; Red: subset of last 300 measured spectra; Green: Full dataset.

Table S20. Pearson correlation coefficients (PCC) among three extracted virus spectra.

Comparison	PCC Value
First vs. Second	0.995
First vs. Full	0.998
Second vs. Full	0.999

The three extracted virus spectra from each dataset exhibits strong similarity. **Table S20** presents the PCC values among the three extracted virus spectra, all of which exceed 0.99. This high correlation indicates that the extracted spectra from different measurements are highly consistent, confirming that our measurement repetitions are sufficient to minimize experimental errors.

S13. The limit of detection (LOD) for virus detection

The limit of detection (LOD) is a critical parameter in spectroscopic analysis, particularly when combined with machine learning techniques for the accurate identification and quantification of viruses. We determined the LOD using the following formulas [24]:

$$LOB = mean_{blank} + 1.645(SD_{blank}) \quad (S2)$$

$$LOD = LOB + 1.645(SD_{low}) \quad (S3)$$

where the limit of blank (LOB) is calculated as the mean of the predicted concentration of the reference samples ($mean_{blank}$), plus 1.645 times the standard deviation of the predicted concentration of these reference samples (SD_{blank}). The multiplier 1.645 corresponds to the 95% confidence interval in a normal distribution, indicating that 95% of reference samples should statistically be distinguishable from those with low concentrations. Subsequently, the LOD is determined by adding 1.645 times the standard deviation of the predicted concentrations of samples with low viral concentrations (SD_{low}). In this study, these "low concentration samples" are defined as those with the lowest possible concentration for each virus. The detailed values of the LOD for each virus can be found in **Table S21**.

Table S21. LOD values of all virus types.

Virus	LOD (PFU/mL)
HMPV-A	23.4
HMPV-B	24.9
CoV-OC43	31.6
Flu B	52.2
CoV-229E	62.2
CoV-NL63	53.0
Ad5	27.3
SARS-CoV-2 B1	16.9
H1N1	46.7
RSV-B1	31.7
H3N2	27.2
RSV-A2	25.2

Table S22. Summary of direct virus detection.

Target virus	SERS substrate	Spectral analysis method	Characterization	LOD	Medium	Ref
Adenovirus<br>Rhinovirus<br>HIV<br>RSV<br>Flu A HKx31 (H3N2)<br>Flu A WSN/33 (H1N1)<br>Flu A PR/8 (H1N1)	AgNR	Calibration curve	Spectral peak identification<br>Quantification	10^3 PFU/mL for RSV	Allantoic fluid<br>Cell lysate<br>others in water	[25]
4 RSV strains	AgNR	PCA, HCA	Classification		Water	[26]
RSV<br>HIV<br>Rotavirus<br>Mycoplasma	AgNR	PCA, HCA	Classification		DMEM<br>PRMI<br>Water	[27]
Avian influenza H5N2	AgNR	PCA, HCA	Classification		Allantoic fluid	[14]
RSV<br>Coxsackievirus B3	Ag coated poly(chloro-*p*-xylylene) film		Spectral peak identification		NaCl, tris-HCl, $MgSO_4$ and sucrose	[28]
8 Rotavirus strains	AgNR	PLS-DA, PLS	Classification<br>Quantification	1.4×10^4 FFU/mL	MA104 cell lysate	[29]
Norovirus MNV4<br>Adenovirus Mad-1<br>Parvovirus MVMp<br>Simian rotavirus SA11<br>Coronavirus MHV<br>Sendai virus Sendai	Klarite^{TM}	PCA	Classification		PBS	[30]
Encephalomyocarditis virus (EMCV)<br>Adenovirus<br>Flu A H1N1	Inverted triangular Au nano-cavities array		Spectral peak identification<br>Quantification	10^6 PFU/mL for EMCV & Adenovirus<br>10^4 PFU/mL for Flu A	DMEM, PBS and cell lysate	[31]
EMCV<br>Adenovirus 5<br>Flu A H1N1<br>Flu A H3N2<br>Flu A H2N2	FIB Au nanorod array		Spectral peak identification<br>SERS substrate optimization		DMEM, PBS and cell lysate	[32]
avian influenza virus (AVI) H5N1<br>AVI H5N2<br>AVI H5N3	AgNR	PLS-DA<br>PCA	Spectral peak identification<br>Classification		Allantoic fluid	[33]
Porcine circovirus 2 (PCV2)<br>Porcine pseudorabies virus (PRV)<br>AVI H5N1	Sputter coated Ag nanoflowers	PCA	Spectral peak identification<br>Classification<br>Quantification	10^3 PFU/mL for PCV2	Water	[34]
Rhinovirus type 4	Electrochemical		Spectral peak			[35]

Poliovirus type 1 Mahoney (PV1M)<br>Poliovirus type 1 Sabin (PV1S)	deposition Ag nanoplates		identification			
PCV2<br>Porcine parvovirus (PPV)<br>PRV	AgNP decorated porous carbon films	PCA	Spectral peak identification<br>Classification	10^7 PFU/mL	Water	36
Myxoma virus (MYXV)<br>Canine Distemper virus (CDV)	Ag thin film (300 nm) on mica	PCA-LDA	Spectral peak identification<br>Classification		tris-HCl solution (pH 8.0)	37
Poliovirus	Au NP clusters-in-bowl array		Spectral peak identification			38
Adenovirus 5<br>Coxsackievirus type 3Cv3	Gold hollow nanocones in microbowls array		Spectral peak identification		PBS	39
hepatitis A virus (HAV)	Au nanopyramid			5260 PFU/mL	Water	40
SARS-CoV-2<br>Flu H1N1 A<br>Marburg<br>Zika virus	Nanoimprinted Ag/SiO_2 multilayered pillars	PCA, RF	Spectral peak identification<br>Classification		Saliva<br>Treated by radiation & heat<br>Cell lysate	41
SARS-CoV-2 pseudovirus<br>Vesicular stomatitis pseudovirus	Gold nanocavities<br>Au $NPs/pZrO_2$ nanobowls		Spectral peak identification<br>Inactivation effect		Cell lysate	42
13 respiratory viruses	SiO_2 coated AgNR	SVM, SVR	Spectral peak identification<br>Classification<br>Quantitation	~ 190 PFU/mL	DMEM<br>Saliva	43
SARS-CoV-2	TiO_2 coated dewetted Au NPs	Ablation-assisted autoencoder	Spectral peak identification<br>Classification<br>Quantitation	10 PFU/mL	Artificial respiratory solution	44
SARS-CoV-2	Ag island film	PLS-DA<br>PCA-LDA<br>SVMC	Spectral peak identification<br>Classification		Saliva<br>Nasal swap	45
HIV-1 X4<br>HIV-1 R5	AgNR, Au coated AgNR	Calibration curve<br>PCA	Spectral peak identification<br>Classification<br>Quantitation	~ 100-200 copies/ml	water	46
Rhinovirus<br>Influenza<br>Parainfluenza	AuNPs on carbon nanotube array	PCA	Classification			47
14 viruses	AuNPs on carbon nanotube array	CNN	Spectral peak identification<br>Classification		Virus cultures	48
Flu-A	Dendritic Ag@c-Si nanostructure	PCA<br>SVM	Spectral peak identification<br>Classification		STE buffer	49
SARS-CoV-2	100 nm g coated Si nanostructures	PCA<br>GNB<br>RF	Spectral peak identification<br>Classification		Saliva and nasal swabs	50

	fabricated by ultrafast laser pulse	SVC<br>LR				
Turnip yellow mosaic Virus (TYMV)	Au NP (29 nm)	PCA-LDA	Spectral peak identification<br>Classification		Sodium phosphate buffer	[51]
Pseudotyped viruses<br>HIV-1<br>Two Flu A H1N1 strains	Au NP (80 nm)		Spectral peak identification			[52]
SARS-CoV-2<br>Human adenovirus type 7<br>Flu A H1N1	Clustered silver NPs (37 nm)	LDA	Spectral peak identification<br>Classification<br>Quantitation	10 PFU/test	Saliva,<br>Serum	[53]
SARS-CoV-2<br>Human adenovirus 3<br>Flu A H1N1	Silver NPs clusters with acetonitrile	PCA	Spectral peak identification<br>Classification<br>Quantitation	100 copies/test (PFU/test)	PBS<br>Saliva,<br>Serum	[54]
Monkeypox virus (MPXV)<br>Human papillomavirus (HPV)	Silver NPs (29 & 40 nm)	PCA	Spectral peak identification<br>Classification<br>Quantitation	100 copies/mL	fetal bovine serum (FBS)<br>artificial vaginal discharge	[55]
SARS-CoV-2<br>SARS-CoV<br>MERS-CoV	AuNPs (50 nm)	PCA<br>SVM	Classification		Saliva	[56]
SARS-CoV-2	SnS_2 microspheres	PCA<br>SVM	Classification			[57]
SARS-CoV-2<br>RSV<br>Flu-B<br>CoV OC43	Si nanostructure made by ultrashort pulsed laser	PCA<br>PLS	Spectral peak identification<br>Classification<br>Quantitation	50 viral particle/ml	Saliva	[58]

References

(1) Piret, J.; Boivin, G. Viral Interference between Respiratory Viruses. *Emerg Infect Dis* **2022**, *28* (2), 273-281. DOI: 10.3201/eid2802.211727 PubMed.

(2) Hendaus, M. A.; Jomha, F. A. Can virus–virus interactions impact the dynamics of the covid-19 pandemic? *Journal of Biomolecular Structure and Dynamics* **2021**, 1-5. DOI: 10.1080/07391102.2021.1926327.

(3) Mahalingam, S.; Schwarze, J.; Zaid, A.; Nissen, M.; Sloots, T.; Tauro, S.; Storer, J.; Alvarez, R.; Tripp, R. A. Perspective on the host response to human metapneumovirus infection: what can we learn from respiratory syncytial virus infections? *Microbes and Infection* **2006**, *8* (1), 285-293. DOI: https://doi.org/10.1016/j.micinf.2005.07.001.

(4) Boyoglu-Barnum, S.; Todd, S. O.; Meng, J.; Barnum, T. R.; Chirkova, T.; Haynes, L. M.; Jadhao, S. J.; Tripp, R. A.; Oomens, A. G.; Moore, M. L.; Anderson, L. J. Mutating the CX3C Motif in the G Protein Should Make a Live Respiratory Syncytial Virus Vaccine Safer and More Effective. *Journal of Virology* **2017**, *91* (10), e02059-02016. DOI: 10.1128/JVI.02059-16 (acccessed 2022/08/24).

(5) Murray, J.; Hogan, R. J.; Martin, D. E.; Blahunka, K.; Sancilio, F. D.; Balyan, R.; Lovern, M.; Still, R.; Tripp, R. A. Probenecid inhibits SARS-CoV-2 replication in vivo and in vitro. *Scientific Reports* **2021**, *11* (1), 18085. DOI: 10.1038/s41598-021-97658-w.

(6) Tripp Ralph, A.; Moore, D.; Jones, L.; Sullender, W.; Winter, J.; Anderson Larry, J. Respiratory Syncytial Virus G and/or SH Protein Alters Th1 Cytokines, Natural Killer Cells, and Neutrophils Responding to Pulmonary Infection in BALB/c Mice. *Journal of Virology* **1999**, *73* (9), 7099-7107. DOI: 10.1128/JVI.73.9.7099-7107.1999 (acccessed 2022/08/24).
(7) Liu, Y. J.; Zhao, Y. P. Simple model for surface-enhanced Raman scattering from tilted silver nanorod array substrates. *Physical Review B* **2008**, *78* (7), 075436. DOI: 10.1103/PhysRevB.78.075436.
(8) Driskell, J. D.; Shanmukh, S.; Liu, Y.; Chaney, S. B.; Tang, X. J.; Zhao, Y. P.; Dluhy, R. A. The Use of Aligned Silver Nanorod Arrays Prepared by Oblique Angle Deposition as Surface Enhanced Raman Scattering Substrates. *The Journal of Physical Chemistry C* **2008**, *112* (4), 895-901. DOI: 10.1021/jp075288u.
(9) Liu, Y. J.; Chu, H. Y.; Zhao, Y. P. Silver Nanorod Array Substrates Fabricated by Oblique Angle Deposition: Morphological, Optical, and SERS Characterizations. *The Journal of Physical Chemistry C* **2010**, *114* (18), 8176-8183. DOI: 10.1021/jp1001644.
(10) Liu, Y. J.; Zhang, Z. Y.; Zhao, Q.; Dluhy, R. A.; Zhao, Y. P. Surface Enhanced Raman Scattering from an Ag Nanorod Array Substrate: The Site Dependent Enhancement and Layer Absorbance Effect. *The Journal of Physical Chemistry C* **2009**, *113* (22), 9664-9669. DOI: 10.1021/jp902142y.
(11) Liu, Y. J.; Chu, H. Y.; Zhao, Y. P. Silver Nanorod Array Substrates Fabricated by Oblique Angle Deposition: Morphological, Optical, and SERS Characterizations. *Journal of Physical Chemistry C* **2010**, *114* (18), 8176-8183. DOI: 10.1021/jp1001644.
(12) Liu, Y.-J.; Zhang, Z.-Y.; Zhao, Q.; Dluhy, R.; Zhao, Y.-P. Surface enhanced Raman scattering from an Ag nanorod array substrate: the site dependent enhancement and layer absorbance effect. *The Journal of Physical Chemistry C* **2009**, *113* (22), 9664-9669.
(13) Song, C.; Chen, J.; Abell, J. L.; Cui, Y.; Zhao, Y. Ag–SiO2 Core–Shell Nanorod Arrays: Morphological, Optical, SERS, and Wetting Properties. *Langmuir* **2012**, *28* (2), 1488-1495. DOI: 10.1021/la203772u.
(14) Abell, J. L.; Driskell, J. D.; Dluhy, R. A.; Tripp, R. A.; Zhao, Y. P. Fabrication and characterization of a multiwell array SERS chip with biological applications. *Biosensors and Bioelectronics* **2009**, *24* (12), 3663-3670. DOI: https://doi.org/10.1016/j.bios.2009.05.039.
(15) Yang, Y.; Xu, B.; Haverstick, J.; Ibtehaz, N.; Muszyński, A.; Chen, X.; Chowdhury, M. E. H.; Zughaier, S. M.; Zhao, Y. Differentiation and classification of bacterial endotoxins based on surface enhanced Raman scattering and advanced machine learning. *Nanoscale* **2022**, *14* (24), 8806-8817, 10.1039/D2NR01277D. DOI: 10.1039/D2NR01277D.
(16) Shapiro, S. S. An analysis of variance test for normality (complete samples). *Biometrika* **1965**, *52*.
(17) Park, K. I. *Fundamentals of Probability and Stochastic Processes with Applications to Communications*; Springer, 2018.
(18) Box, G. E. P. P., D. A. . Distribution of Residual Autocorrelations in Autoregressive-Integrated Moving Average Time Series Models. *Journal of the American Statistical Association* **1970**, *65*.
(19) Box, G. M. L. G. E. P. On a Measure of a Lack of Fit in Time Series Models. *Biometrika* **1978**, *65*.
(20) Zhu, J.; Sharma, A. S.; Xu, J.; Xu, Y.; Jiao, T.; Ouyang, Q.; Li, H.; Chen, Q. Rapid on-site identification of pesticide residues in tea by one-dimensional convolutional neural network coupled with surface-enhanced Raman scattering. *Spectrochimica Acta Part A: Molecular and Biomolecular Spectroscopy* **2021**, *246*, 118994. DOI: https://doi.org/10.1016/j.saa.2020.118994.
(21) Fan, X., Ming, W., Zeng, H., Zhang, Z., & Lu, H. Deep learning-based component identification for the Raman spectra of mixtures. *The Analyst* **2019**, *144*.
(22) Cortes, C. a. V., Vladimir. Support-vector networks. *Machine Learning* **1995**, *20*, 273--297. DOI: 10.1007/BF00994018.
(23) Ho, T. K. Random Decision Forests. In *Proceedings of 3rd International Conference on Document Analysis and Recognition*, Vol. 1; 1995; pp 278--282.
(24) Armbruster, D. A.; Pry, T. J. T. c. b. r. Limit of blank, limit of detection and limit of quantitation. *Clinical Biochemist Reviews* **2008**, *29* (Suppl 1), S49.
(25) Shanmukh, S.; Jones, L.; Driskell, J.; Zhao, Y.; Dluhy, R.; Tripp, R. A. Rapid and Sensitive Detection of Respiratory Virus Molecular Signatures Using a Silver Nanorod Array SERS Substrate. *Nano Letters* **2006**, *6* (11), 2630-2636. DOI: 10.1021/nl061666f.
(26) Shanmukh, S.; Jones, L.; Zhao, Y. P.; Driskell, J. D.; Tripp, R. A.; Dluhy, R. A. Identification and classification of respiratory syncytial virus (RSV) strains by surface-enhanced Raman spectroscopy and multivariate statistical techniques. *Analytical and Bioanalytical Chemistry* **2008**, *390* (6), 1551-1555. DOI: 10.1007/s00216-008-1851-0.

(27) Driskell, J. D.; Shanmukh, S.; Liu, Y.-J.; Hennigan, S.; Jones, L.; Zhao, Y.-P.; Dluhy, R. A.; Krause, D. C.; Tripp, R. A. Infectious agent detection with SERS-active silver nanorod arrays prepared by oblique angle deposition. *Ieee Sensors Journal* **2008**, *8* (5-6), 863-870. DOI: 10.1109/jsen.2008.922682.
(28) Demirel, M. C.; Kao, P.; Malvadkar, N.; Wang, H.; Gong, X.; Poss, M.; Allara, D. L. Bio-organism sensing via surface enhanced Raman spectroscopy on controlled metal/polymer nanostructured substrates. *Biointerphases* **2009**, *4* (2), 35-41. DOI: 10.1116/1.3147962.
(29) Driskell, J. D.; Zhu, Y.; Kirkwood, C. D.; Zhao, Y.; Dluhy, R. A.; Tripp, R. A. Rapid and Sensitive Detection of Rotavirus Molecular Signatures Using Surface Enhanced Raman Spectroscopy. *PLoS One* **2010**, *5* (4), e10222. DOI: 10.1371/journal.pone.0010222.
(30) Fan, C.; Hu, Z.; Riley, L. K.; Purdy, G. A.; Mustapha, A.; Lin, M. Detecting food- and waterborne viruses by surface-enhanced Raman spectroscopy. *J Food Sci* **2010**, *75* (5), M302-307. DOI: 10.1111/j.1750-3841.2010.01619.x From NLM.
(31) Chang, C.-W.; Liao, J.-D.; Shiau, A.-L.; Yao, C.-K. Non-labeled virus detection using inverted triangular Au nano-cavities arrayed as SERS-active substrate. *Sensors and Actuators B: Chemical* **2011**, *156* (1), 471-478. DOI: https://doi.org/10.1016/j.snb.2011.04.006.
(32) Lin, Y.-Y.; Liao, J.-D.; Yang, M.-L.; Wu, C.-L. Target-size embracing dimension for sensitive detection of viruses with various sizes and influenza virus strains. *Biosensors and Bioelectronics* **2012**, *35* (1), 447-451. DOI: https://doi.org/10.1016/j.bios.2012.02.041.
(33) Chunyuan, S.; Jeremy, D. D.; Ralph, A. T.; Yiping, C.; Yiping, Z. The use of a handheld Raman system for virus detection. In *Proc.SPIE*, 2012; Vol. 8358, p 83580I. DOI: 10.1117/12.918758.
(34) Shao, F.; Lu, Z.; Liu, C.; Han, H.; Chen, K.; Li, W.; He, Q.; Peng, H.; Chen, J. Hierarchical Nanogaps within Bioscaffold Arrays as a High-Performance SERS Substrate for Animal Virus Biosensing. *ACS Applied Materials & Interfaces* **2014**, *6* (9), 6281-6289. DOI: 10.1021/am4045212.
(35) Yang, S.; Slotcavage, D.; Mai, J. D.; Guo, F.; Li, S.; Zhao, Y.; Lei, Y.; Cameron, C. E.; Huang, T. J. Electrochemically created highly surface roughened Ag nanoplate arrays for SERS biosensing applications. *Journal of Materials Chemistry C* **2014**, *2* (39), 8350-8356. DOI: 10.1039/c4tc01276c.
(36) Luo, Z.; Chen, L.; Liang, C.; Wei, Q.; Chen, Y.; Wang, J. Porous carbon films decorated with silver nanoparticles as a sensitive SERS substrate, and their application to virus identification. *Microchimica Acta* **2017**, *184* (9), 3505-3511. DOI: 10.1007/s00604-017-2369-y.
(37) Durmanov, N. N.; Guliev, R. R.; Eremenko, A. V.; Boginskaya, I. A.; Ryzhikov, I. A.; Trifonova, E. A.; Putlyaev, E. V.; Mukhin, A. N.; Kalnov, S. L.; Balandina, M. V.; et al. Non-labeled selective virus detection with novel SERS-active porous silver nanofilms fabricated by Electron Beam Physical Vapor Deposition. *Sensors and Actuators B: Chemical* **2018**, *257*, 37-47. DOI: https://doi.org/10.1016/j.snb.2017.10.022.
(38) Wang, Y.; Yu, Y.; Liu, Y.; Yang, S. Template-Confined Site-Specific Electrodeposition of Nanoparticle Cluster-in-Bowl Arrays as Surface Enhanced Raman Spectroscopy Substrates. *ACS Sensors* **2018**, *3* (11), 2343-2350. DOI: 10.1021/acssensors.8b00711.
(39) Zhang, X.; Zhang, X.; Luo, C.; Liu, Z.; Chen, Y.; Dong, S.; Jiang, C.; Yang, S.; Wang, F.; Xiao, X. Volume-Enhanced Raman Scattering Detection of Viruses. *Small* **2019**, *15* (11), 1805516, https://doi.org/10.1002/smll.201805516. DOI: https://doi.org/10.1002/smll.201805516 (acccessed 2022/07/12).
(40) Palermo, G.; Rippa, M.; Conti, Y.; Vestri, A.; Castagna, R.; Fusco, G.; Suffredini, E.; Zhou, J.; Zyss, J.; De Luca, A.; Petti, L. Plasmonic Metasurfaces Based on Pyramidal Nanoholes for High-Efficiency SERS Biosensing. *ACS Applied Materials & Interfaces* **2021**, *13* (36), 43715-43725. DOI: 10.1021/acsami.1c12525.
(41) Paria, D.; Kwok, K. S.; Raj, P.; Zheng, P.; Gracias, D. H.; Barman, I. Label-Free Spectroscopic SARS-CoV-2 Detection on Versatile Nanoimprinted Substrates. *Nano Letters* **2022**, *22* (9), 3620-3627. DOI: 10.1021/acs.nanolett.1c04722.
(42) Sitjar, J.; Xu, H.-Z.; Liu, C.-Y.; Wang, J.-R.; Liao, J.-D.; Tsai, H.-P.; Lee, H.; Liu, B. H.; Chang, C.-W. Synergistic surface-enhanced Raman scattering effect to distinguish live SARS-CoV-2 S pseudovirus. *Analytica Chimica Acta* **2022**, *1193*, 339406. DOI: https://doi.org/10.1016/j.aca.2021.339406.
(43) Yang, Y.; Xu, B.; Murray, J.; Haverstick, J.; Chen, X.; Tripp, R. A.; Zhao, Y. Rapid and quantitative detection of respiratory viruses using surface-enhanced Raman spectroscopy and machine learning. *Biosensors and Bioelectronics* **2022**, *217*, 114721. DOI: https://doi.org/10.1016/j.bios.2022.114721.
(44) Hwang, C. S. H.; Lee, S.; Lee, S.; Kim, H.; Kang, T.; Lee, D.; Jeong, K.-H. Highly Adsorptive Au-TiO2 Nanocomposites for the SERS Face Mask Allow the Machine-Learning-Based Quantitative Assay of SARS-CoV-2 in Artificial Breath Aerosols. *ACS Applied Materials & Interfaces* **2022**, *14* (49), 54550-54557. DOI:

10.1021/acsami.2c16446.
(45) Berus, S. M.; Nowicka, A. B.; Wieruszewska, J.; Niciński, K.; Kowalska, A. A.; Szymborski, T. R.; Dróżdż, I.; Borowiec, M.; Waluk, J.; Kamińska, A. SERS Signature of SARS-CoV-2 in Saliva and Nasopharyngeal Swabs: Towards Perspective COVID-19 Point-of-Care Diagnostics. In *International Journal of Molecular Sciences*, 2023; Vol. 24.
(46) Yadav, S.; Senapati, S.; Kulkarni, S. S.; Singh, J. P. A SERS based clinical study on HIV-1 viral load quantification and determination of disease prognosis. *Journal of Photochemistry and Photobiology B: Biology* **2023**, *239*, 112629. DOI: https://doi.org/10.1016/j.jphotobiol.2022.112629.
(47) Yeh, Y.-T.; Gulino, K.; Zhang, Y.; Sabestien, A.; Chou, T.-W.; Zhou, B.; Lin, Z.; Albert, I.; Lu, H.; Swaminathan, V.; et al. A rapid and label-free platform for virus capture and identification from clinical samples. *Proceedings of the National Academy of Sciences* **2020**, *117* (2), 895-901.
(48) Ye, J.; Yeh, Y.-T.; Xue, Y.; Wang, Z.; Zhang, N.; Liu, H.; Zhang, K.; Ricker, R.; Yu, Z.; Roder, A.; et al. Accurate virus identification with interpretable Raman signatures by machine learning. *Proceedings of the National Academy of Sciences* **2022**, *119* (23), e2118836119. DOI: 10.1073/pnas.2118836119 (acccessed 2022/10/12).
(49) Prigoda, K.; Ermina, A.; Bolshakov, V.; Tabarov, A.; Levitskii, V.; Andreeva, O.; Gazizulin, A.; Pavlov, S.; Danilenko, D.; Vitkin, V.; Zharova, Y. Dendritic Ag@c-Si structure for influenza A virus detection by SERS and machine learning. *Optical Materials* **2024**, *149*, 114977. DOI: https://doi.org/10.1016/j.optmat.2024.114977.
(50) Szymborski, T. R.; Berus, S. M.; Nowicka, A. B.; Słowiński, G.; Kamińska, A. Machine Learning for COVID-19 Determination Using Surface-Enhanced Raman Spectroscopy. In *Biomedicines*, 2024; Vol. 12.
(51) Kim, S.; Lee, S.; Chi, H. Y.; Kim, M. K.; Kim, J. S.; Lee, S. H.; Chung, H. Feasibility Study for Detection of Turnip yellow mosaic virus (TYMV) Infection of Chinese Cabbage Plants Using Raman Spectroscopy. *Plant Pathol J* **2013**, *29* (1), 105-109. DOI: 10.5423/ppj.Nt.09.2012.0147 From NLM.
(52) Lim, J.-y.; Nam, J.-s.; Yang, S.-e.; Shin, H.; Jang, Y.-h.; Bae, G.-U.; Kang, T.; Lim, K.-i.; Choi, Y. Identification of Newly Emerging Influenza Viruses by Surface-Enhanced Raman Spectroscopy. *Analytical Chemistry* **2015**, *87* (23), 11652-11659. DOI: 10.1021/acs.analchem.5b02661.
(53) Zhang, Z.; Jiang, S.; Wang, X.; Dong, T.; Wang, Y.; Li, D.; Gao, X.; Qu, Z.; Li, Y. A novel enhanced substrate for label-free detection of SARS-CoV-2 based on surface-enhanced Raman scattering. *Sensors and Actuators B: Chemical* **2022**, *359*, 131568. DOI: https://doi.org/10.1016/j.snb.2022.131568.
(54) Zhang, Z.; Li, D.; Wang, X.; Wang, Y.; Lin, J.; Jiang, S.; Wu, Z.; He, Y.; Gao, X.; Zhu, Z.; et al. Rapid detection of viruses: Based on silver nanoparticles modified with bromine ions and acetonitrile. *Chemical Engineering Journal* **2022**, *438*, 135589. DOI: https://doi.org/10.1016/j.cej.2022.135589.
(55) Lv, X. P.; Zhang, Z.; Zhao, Y.; Sun, X. M.; Jiang, H.; Zhang, S. W.; Sun, X. Q.; Qiu, X. H.; Li, Y. Label-free detection of virus based on surface-enhanced Raman scattering. *Spectrochimica Acta Part a-Molecular and Biomolecular Spectroscopy* **2023**, *302*. DOI: 10.1016/j.saa.2023.123087.
(56) Karunakaran, V.; Joseph, M. M.; Yadev, I.; Sharma, H.; Shamna, K.; Saurav, S.; Sreejith, R. P.; Anand, V.; Beegum, R.; Regi David, S.; et al. A non-invasive ultrasensitive diagnostic approach for COVID-19 infection using salivary label-free SERS fingerprinting and artificial intelligence. *Journal of Photochemistry and Photobiology B: Biology* **2022**, *234*, 112545. DOI: https://doi.org/10.1016/j.jphotobiol.2022.112545.
(57) Peng, Y.; Lin, C.; Li, Y.; Gao, Y.; Wang, J.; He, J.; Huang, Z.; Liu, J.; Luo, X.; Yang, Y. Identifying infectiousness of SARS-CoV-2 by ultra-sensitive SnS2 SERS biosensors with capillary effect. *Matter* **2022**, *5* (2), 694-709. DOI: https://doi.org/10.1016/j.matt.2021.11.028.
(58) Ganesh, S.; Dhinakaran, A. K.; Premnath, P.; Venkatakrishnan, K.; Tan, B. Label-Free Saliva Test for Rapid Detection of Coronavirus Using Nanosensor-Enabled SERS. In *Bioengineering*, 2023; Vol. 10.
